# Anisotropic forces from spatially constrained focal adhesions mediate contact guidance directed cell migration

**DOI:** 10.1038/ncomms14923

**Published:** 2017-04-12

**Authors:** Arja Ray, Oscar Lee, Zaw Win, Rachel M. Edwards, Patrick W. Alford, Deok-Ho Kim, Paolo P. Provenzano

**Affiliations:** 1 Department of Biomedical Engineering, University of Minnesota, Minneapolis, Minnesota 55455, USA; 2 University of Minnesota Physical Sciences in Oncology Center, Minneapolis, Minnesota 55455, USA; 3 Department of Bioengineering, University of Washington, Seattle, Washington 98105, USA; 4 Institute for Engineering in Medicine, University of Minnesota, Minneapolis, Minnesota 55455, USA; 5 Masonic Cancer Center, University of Minnesota, Minneapolis, Minnesota 55455, USA; 6 Stem Cell Institute, University of Minnesota, Minneapolis, Minnesota 55455, USA

## Abstract

Directed migration by contact guidance is a poorly understood yet vital phenomenon, particularly for carcinoma cell invasion on aligned collagen fibres. We demonstrate that for single cells, aligned architectures providing contact guidance cues induce constrained focal adhesion maturation and associated F-actin alignment, consequently orchestrating anisotropic traction stresses that drive cell orientation and directional migration. Consistent with this understanding, relaxing spatial constraints to adhesion maturation either through reduction in substrate alignment density or reduction in adhesion size diminishes the contact guidance response. While such interactions allow single mesenchymal-like cells to spontaneously ‘sense' and follow topographic alignment, intercellular interactions within epithelial clusters temper anisotropic cell–substratum forces, resulting in substantially lower directional response. Overall, these results point to the control of contact guidance by a balance of cell–substratum and cell–cell interactions, modulated by cell phenotype-specific cytoskeletal arrangements. Thus, our findings elucidate how phenotypically diverse cells perceive ECM alignment at the molecular level.

## Abstract (web summary)

Contact guidance on aligned substrates leads to directed cell migration through a poorly defined mechanism. Here the authors show that alignment of adhesion structures and F-actin generates anisotropic traction stress to drive directional migration, and cell-cell contact reduces force orientation and directional response.

It is well established that cell migration, using diverse phenotypic modes (for example, collective, mesenchymal, amoeboid), is not only essential for normal processes such as embryonic development, immune function and tissue repair, but also plays a critical role in disease states including cancer dissemination^1^,^2^,^3^,^4^,^5^. However, our understanding of the molecular and physical mechanisms that regulate cell migration in normal and pathologic states remains incomplete. In particular, the mechanisms governing directional migration, a critical process whereby cells migrate with high persistence on a path, is not well understood. Directional migration, in contradistinction to random cell motility^6^, can be established either through intrinsic cellular mechanisms or driven by single, or combinations of, external cues such as chemical gradients (that is, chemotaxis), extracellular matrix (ECM) adhesion sites or substrate-bound chemoattractant gradients (that is, haptotaxis), gradients in substrate stiffness (that is, durotaxis) or the tendency of cells to migrate along anisotropic, aligned, structures (that is, contact guidance)^7^,^8^,^9^,^10^.

Contact guidance where cells utilize anisotropy, often in the form of aligned ECM fibres to orient and migrate along single or multiple fibres is indeed a strong regulator of directed migration that is implicated in numerous developmental, physiologic, and pathophysiologic processes^11^,^12^,^13^. In fact, it is a strong regulator of carcinoma progression^14^,^15^,^16^,^17^. For instance, we characterized unique collagen architectures in the desmoplastic tumour stroma that promote focal invasion and metastasis^16^, influence disease state and progression dynamics^15^,^18^,^19^ and correlate with significantly worse survival in human patients^17^. In that context, we identified a panel of tumour-associated collagen signatures, or TACS, which provide standard hallmarks to locate and characterize tumors^14^,^15^,^16^. Included in this series is TACS-3 where collagen fibres are aligned and reorganized perpendicular to breast carcinoma cell cluster boundaries within and around the tumour mass^16^ to promote contact guidance-mediated migration and focal invasion^16^,^20^. Further, it is becoming clear that these alignment patterns are not unique to breast cancer, as recent evidence suggests that pancreatic ductal adenocarcinoma (PDA), another highly metastatic desmoplastic disease, is rife with TACS-3-like architectures^21^,^22^.

Despite a critical role in health and disease, the physical and molecular mechanisms governing directional migration by contact guidance have defied elucidation. Although several reports exist in the literature alluding to the fact that cells respond to ECM alignment, a close examination suggested to us that the degree to which different cell types respond to topographic alignment may vary^23^,^24^,^25^. However in the absence of a single, comprehensive study comparing different cell types under the same substratum conditions, it is difficult to make decisive conclusions. Although a handful of studies compare the responses of phenotypically different cells to topographic alignment^26^,^27^,^28^, none, to our knowledge have comprehensively addressed this issue in cancer cells, which are known to be a heterogeneous mix displaying diverse modes of migration^6^, thus making such insights particularly relevant. In addition, apart from a few key studies (for example, refs 29, 30), disproportionately little work has sought to comprehensively answer the fundamental question: how do cells spontaneously sense topographic alignment at the molecular level?

Here, we address these knowledge gaps and identify physical and molecular mechanisms governing how cells sense and respond to aligned ECM architectures. We find that the anisotropy in substratum structure physically constrains focal adhesion (FA) maturation, thereby spontaneously translating matrix alignment to cellular guidance through anisotropic forces. Thus, force generation and therefore F-actin organization associated with adhesion complexes are key elements modulating contact guidance that can be perturbed to diminish the contact guidance response. Further, different cell types, with their unique FA-cytoskeletal arrangements thus respond differently to the same contact guidance cue. Most notably, we find that cell–cell contacts restrain contact guidance by counteracting the anisotropic cell–ECM interactions. Together, our findings mechanistically explicate the observed diversity in responses to contact guidance across cell types and provide a biophysical perspective to understand and disrupt the directional guidance of carcinoma cells invading on aligned collagen fibres.

## Results

### Contact guidance cues guide dissemination of carcinoma cells

**Figure 1:**
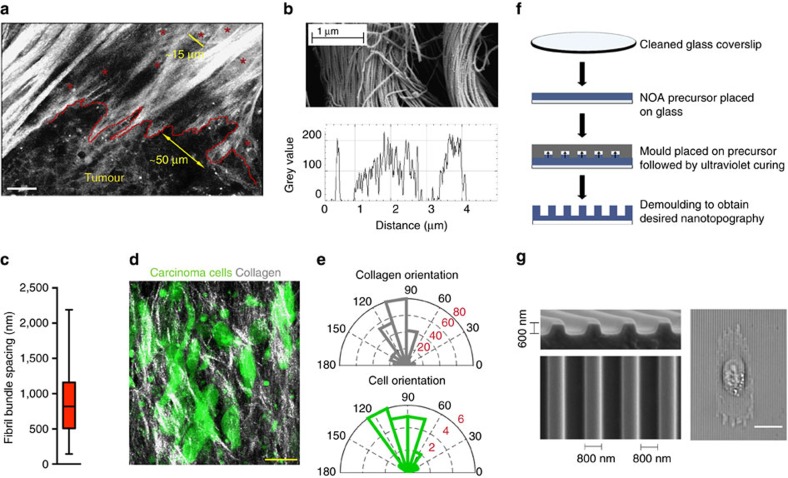
Nanopatterned substrates mimic aligned collagen architectures in breast and pancreas cancer. (**a**) Combined multiphoton excitation (MPE) and SHG image from live mammary carcinoma adapted from Provenzano *et al*.^16^, with permission, demonstrating the distinct cell–ECM interactions present during carcinoma cell invasion into regions of aligned stromal collagen. Asterisks signify single cells that have migrated past the tumour boundary along aligned collagen fibres. Scale bar, 25 μm. (**b**) SEM image of collagen fibrils within mammary collagen fibres and line scan data demonstrating the spacing between fibril bundles. (**c**) Quantification of the mean and full range (bars) of spacings between fibril bundles. (**d**) Combined MPE and SHG imaging showing aligned collagen patterns (grey) interacting with ZsGreen expressing PDA cells (green) within a pancreas tumour. Scale bar, 50μm. (**e**) Angular histogram of orientation of collagen and carcinoma cells from the image in **d** showing cell elongation in the direction of collagen alignment. (**f**) Step-wise methodology for nanofabrication of substrates to model ECM alignment. (**g**) Scanning electron micrograph of the standard aligned substrate possessing ridge and groove widths of 800 nm and ridges that are 600 nm in depth and a micrograph showing a typical MDA-MB-231 cell on a typical aligned substrate oriented along the direction of ECM alignment. Scale bar, 20 μm.

Analysis of combined multiphoton excitation (MPE) and second harmonic generation (SHG) imaging data of carcinoma cells interacting with aligned collagen at the tumour–stroma boundary reveals phenotypically distinct cancer cells ranging from clustered, classically epithelial to single, mesenchymal-like cells associated with the anisotropic ECM (Fig. 1a). Cells undergoing collective migration into the stroma are often bounded by groups of aligned collagen fibres separated by micrometre-scale lengths, consistent with our recent work demonstrating that micrometre-scale spatial confinement is a strong determinant of directed collective migration^31^, while single cells are often directly bounded by aligned collagen fibres (Fig. 1a). However, as expected, ultrastructural analyses of mammary collagen shows that the single, micrometre-scale collagen fibres are in fact bundles of long filamentous fibrils with diameters of 10–100 nm^16^,^32^. Thus, invading carcinoma cells migrating within, between or along collagen fibres interact with the filamentous topography on fibrils or groups of fibrils that are spaced hundreds of nanometres to a few micrometres apart, with a mean spacing of ∼850 nm between fibril bundles (Fig. 1b,c). Furthermore, MPE/SHG imaging of live pancreatic carcinomas in the *KPC* mouse model of PDA with a fluorescent reporter expressed specifically in carcinoma cells displays frequent collagen alignment throughout the stroma (Fig. 1d). Indeed, data in this model supports earlier finding as cells in aligned regions are tightly oriented and closely follow the anisotropy of ECM organization (Fig. 1e). Thus, similar to breast cancer, collagen alignment likely impacts PDA dissemination by contact guidance.

To dissect the physical and molecular determinants of the response of carcinoma cells, we employed nanofabrication technology to generate a platform to impart directional cues that offers a useful compromise between two-dimensional (2D) and three-dimensional (3D) environments, allowing us to perform robust quantitative analysis under defined conditions. To model topographic alignment, we collagen-coated nanopatterned substrates comprised of long parallel ridges and grooves and generated corresponding flat substrates as control environments (Fig. 1f). For most of our studies, we used substrates with 800 nm ridge and groove widths (that is, 1:1 ridge:groove width ratio or pattern density) to mimic mean spacing between fibril bundles (Fig. 1g). However, we perturbed the groove and/or ridge width for certain experiments to range from hundreds of nanometres to a few micrometres, thus mimicking the collagen fibril and fibre spacing dimensions observed *in vivo* (Fig. 1b,c).

### Protrusion directionality during contact guidance

**Figure 2:**
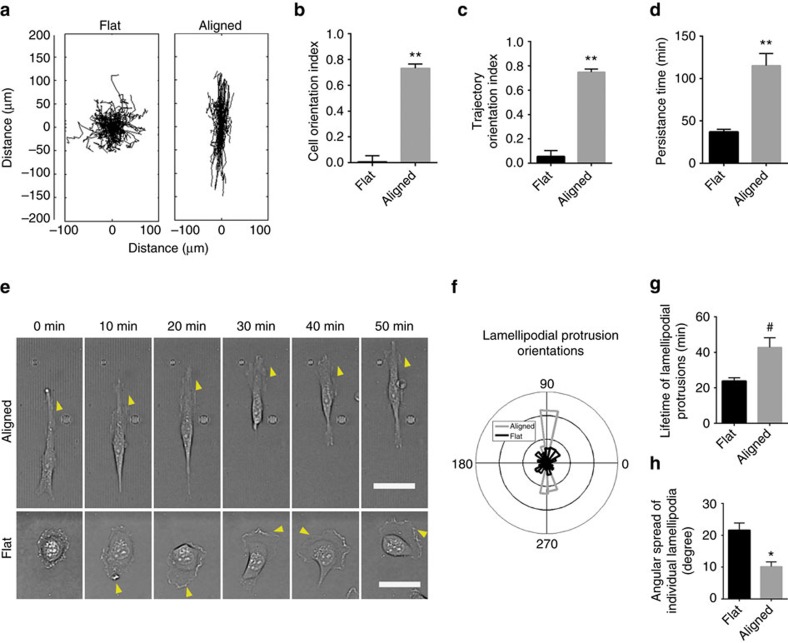
Stable membrane protrusions highly constrained to the direction of ECM alignment are associated with contact guidance-directed migration. (**a**) Migration trajectory map of MDA-MB-231 cells on flat and topographically aligned substrates. (**b**,**c**) Cell orientation (**b**) and Trajectory orientation (**c**) indices for MDA-MB-231 cells on flat versus nanopatterned substrates showing significantly greater directed migration due to contact guidance (*n*>75 cells per group). (**d**) Persistence time of MDA-MB-231 cells on flat and aligned substrates (*n*>85 cell per group). (**e**) Timelapse montage of lamellipodia dynamics over time for MDA-MB-231 cells on flat versus aligned substrates. Arrowheads highlight protrusion stability on aligned ECM or frequent change in direction for cells on flat substrates. Scale bar, 50 μm. (**f**) Wind-rose plot of MDA-MB-231 lamellipodia protrusion orientations showing highly directed protrusion dynamics for cells on aligned ECM (*n*>400 lamellipodia per group). (**g**,**h**) Lamellipodia lifetime (**g**) and Angular spread (**h**) for MDA-MB-231 cells on flat versus aligned substrates demonstrating significantly greater protrusion stability and directionality for cells on aligned ECM (**g**: *n*>75 per group; **h**: *n*=28 per group). Data in **b**–**d**,**f**–**h** are mean±s.e.m. **P<0.0001, *P<0.01, ^#^*P*<0.001 (unpaired *t*-test with Welch's correction: **b**–**d**,**g**; Mann–Whitney test: **h**).

To investigate the operant cellular dynamics during contact guidance of carcinoma cells, we first plated highly metastatic MDA-MB-231 breast cancer cells on flat and aligned collagen-coated substrates and characterized their spreading and migration. The striking contrast in behaviour under these conditions is exemplified by their spontaneous and rapid switch from random to directed migration on encountering aligned topography (Supplementary Movie 1, Fig. 2a). To define this response we quantified cell orientations using a cell orientation index (COI) metric, which assumes a value of 0 for a population of randomly oriented cells and 1 for cells that are perfectly aligned to the patterned substrate. Likewise, cell trajectory orientation analysis was performed using a metric (trajectory orientation index) that captures a normalized, average angular deviation of the trajectories from the alignment direction, again ranging from 0 to 1 for a randomly migrating population and a perfectly aligned trajectory, respectively. On the aligned patterns, MDA-MB-231 cells were more elongated (Supplementary Fig. 1a) and highly oriented along the direction of alignment (Fig. 2b). In addition they showed a >10-fold increase in trajectory orientation (Fig. 2a,c) and a 2.5-fold increase in persistence time (Fig. 2d), the latter being a measure of the length of time cells travel before changing direction, obtained by fitting a persistent random walk model (PRWM) to cell trajectories^8^,^33^. Thus, cell trajectories are straighter and highly directional on the aligned cues versus flat surfaces. The robust response of these breast carcinoma cells that have undergone an epithelial-to-mesenchymal transition (EMT) is consistent with previous reports on contact guidance in fibroblasts in 2D (ref. 23) and 3D (ref. 10), thus making them an ideal cell line for in-depth investigation into the mechanisms governing the contact guidance response.

Analysis of MDA-MB-231 cells encountering aligned and flat topographic features reveals differences in spatial and temporal membrane protrusion dynamics (Fig. 2e, Supplementary Movie 1). Membrane protrusions at the cell's leading edge, termed lamellipodia, influence migration direction and speed largely through adhesion and cytoskeletal dynamics^34^,^35^,^36^. Further, orientation and stability of lamellipodia regulate chemotaxis-directed migration^8^,^37^ as well as high persistence on narrow (termed 1D) ECM tracks with limited lateral cell spreading^38^. Here, contact guidance by submicrometre-scale cues induce lamellipodia tightly constrained to the direction of alignment (Fig. 2f), in agreement with a recent study in aligned 3D collagen matrices^39^. In addition, protrusions on aligned patterns are significantly more stable than those on flat surfaces (Fig. 2g), and for a given protrusion, the distribution of its orientations over time is confined to a much narrower range (Fig. 2h). Thus, the stability and limited angular mobility of lamellipodia appear to drive the highly linear and directional migration trajectories (Fig. 2c,d). To validate these findings, we quantified behaviour in a second (pancreatic) carcinoma line with an EMT phenotype (MIA-PaCa-2) and observed a robust contact guidance response accompanied with similar spatially constrained lamellipodia formation (Supplementary Fig. 1b,c). Further, it is critical to recognize that unlike migration of cells on thin lines of ECM^38^ or in narrow channels^40^, the lamellipodial constraint in this case is induced in the absence of any imposed lateral confinement, as presumably can be the case when cells interact with native aligned fibrillar ECM. Thus, collectively, these results suggest that stable and spatially directed membrane protrusions are strongly associated with the cellular sensing of contact guidance cues and raise interesting questions about the underlying adhesion dynamics regulating the generation and dynamics of these protrusions.

### Physical confinement drives orientation of FAs

**Figure 3:**
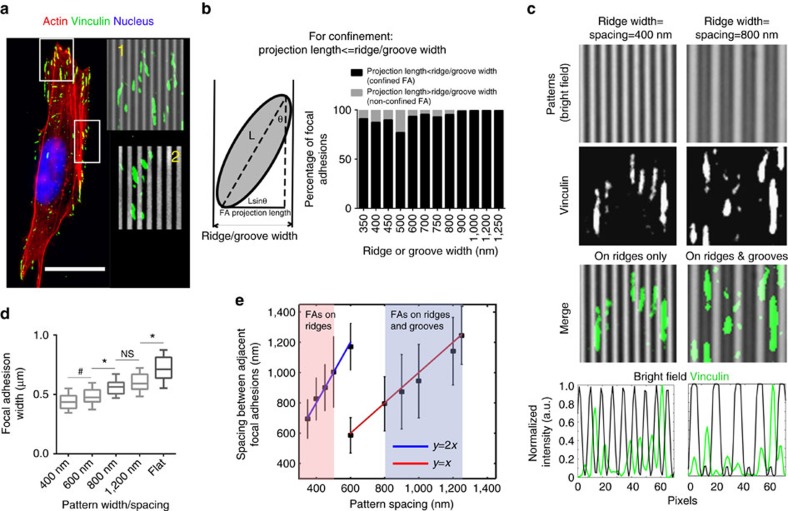
Spatial constraints from aligned topographies direct focal adhesion orientation as well as elongation and biased distribution. (**a**) Representative fluorescence micrograph showing a typical MDA-MB-231 cell aligned on a nanopatterned substrate stained for actin (red), Vinculin (green) and nucleus (blue); scale bar, 20 μm. (inset-1 for top box) Magnified region demonstrating high FA alignment and magnified side region (inset-2 from side box) showing non-aligned Vinculin-stained FA complexes (green) overlaid on the nanopatterns from bright-field image (grey). (**b**) Schematic diagram showing the projection length of an ellipsoidal FA complex confined within a nanopattern ridge or groove and analysis of FA complexes on patterns of varying ridge and groove widths, where the ridge and groove width dimensions are equal for each condition (that is, 1:1 ratio), demonstrating that the vast majority of FAs have projection lengths less than the corresponding pattern dimensions. (**c**) Overlaid bright-field micrographs of nanopatterns (grey) and fluorescence micrographs of Vinculin (green) showing typical adhesion complex distribution on patterns with 1:1 ridge=groove width (for example, 400 nm:400 nm on the left and 800 nm:800 nm on the right) with average line intensities for each along the corresponding abscissa demonstrating FA confinement to individual ridges and grooves. (**d**) Widths of large, aligned FA complexes at the leading edge of cells on aligned substrates increase with increasing pattern widths and are higher still on flat substrates (*n*>65 per group). (**e**) Peak-to-peak distances or spacing between adjacent FAs as a function of pattern width/spacing highlighting the discrete arrangement of FA positions. Pink and blue highlighted regions respectively denote pattern width ranges where adjacent FAs form on consecutive ridges only and on both ridges and grooves, as inferred from the slope of their linear relationship to the pattern widths. Data are median with 10–90th percentile range (**d**) and mean±s.d. (**e**). ^#^*P*<0.05, *P<0.001, NS=no significance (ANOVA).

To elucidate the mechanisms by which carcinoma cells ‘sense' topographic alignment to establish and maintain directional protrusions, we analysed the morphology and distribution of FAs, which are key sensory complexes involved in adhesion and mechanotransduction. For high-throughput analysis of FAs, we developed a semi-automated system integrating CellProfiler and Fiji to identify and measure morphological features of FA complexes from vinculin-stained fluorescence micrographs (Supplementary Fig. 2a). Scrutinizing the morphologies of populations of single FAs as a function of defined substrate architectures, we found that the majority of large, oriented and highly elongated adhesions are found abundantly at the front and rear of an aligned cell, while the smaller adhesions are primarily localized laterally (Fig. 3a). Examination of these adhesions overlaid with bright-field images led to the striking observation that adhesion complexes are bounded by the topographic discontinuities, that is, an adhesion complex on a ridge/groove only spans the space within the boundary of that ridge/groove (Fig. 3a). Thus, while an adhesion can mature unhindered along the direction of aligned ECM, their growth in the orthogonal direction is restricted by the pattern width. This is consistent with live cell imaging data of single GFP-paxillin expressing MDA-MB-468 (another triple negative breast carcinoma line) cells at the boundary of flat and aligned topographies demonstrating that as the cell moves into the aligned region, one can clearly observe large, non-aligned adhesions reorganize into confined, aligned adhesions as they encounter the substrate architecture (Supplementary Movie 2). Furthermore, to confirm these observations we investigated FA morphology on patterns of varying ridge and groove width from 350 to 1,250 nm (1:1 ridge=groove spacing for each; Supplementary Fig. 2b). A necessary condition for FA confinement to individual ridges/grooves is that the projection length of any particular FA be less than the ridge or groove width for the underlying pattern (Fig. 3b). Indeed, we found that this condition holds for the vast majority of FAs across different ridge and groove widths, that is, ∼90+% of FAs have projection lengths that do not exceed the width of the ridge or groove they form on (Fig. 3b). These results are in agreement with a model for contact-guided endothelial cell spreading by Franco *et al*.^30^. Likewise, a recent study by Horwitz and co-workers suggests that in 3D matrices, individual adhesion size is limited by collagen fibre thickness^41^, thereby indicating that our findings are likely translatable to other cell types and more complex 3D environments.

Superposing bright-field intensities with vinculin staining clearly shows that adhesions are narrowly confined within a single ridge or a groove (Fig. 3c). Due to these boundary conditions, parallel FA arrays in lamellipodia form unique spatial arrangements that can be quantified by the peak-to-peak distance, or spacing, between adjacent adhesions (Fig. 3c). On patterns with spacing <550 nm, the spacing between contiguous adhesion complexes is twice that of the pattern spacing, indicating that adhesions form on successive ridges only, and not on adjacent grooves between the ridges (Fig. 3c (left panel) and Fig. 3e, Supplementary Fig. 2c). On the other hand, for pattern spacing ≥700 nm, there exists a one-to-one correlation between pattern spacing and the gap between adjacent FAs, demonstrating that adhesions form on both ridges and grooves (Fig. 3c (right panel), Fig. 3e, Supplementary Fig. 2c). In fact, at 600 nm spacing, the distribution of FA spacing is bimodal, representing a switching point between the two regimes (Fig. 3e, Supplementary Fig. 2c). Further, physical constraints of the topography limit FA growth in lateral directions leading to directional maturation and orientation of FAs along substratum guidance cues. Thus, the width of large FAs formed at the leading edge of cells is limited by submicrometre pattern widths and therefore increases with the latter, assuming a maximum value when unconstrained on a flat substrate (Fig. 3d). In contrast, the corresponding average adhesion lengths at the leading edge ranged from ∼2.5 to 3 μm and were higher on the aligned substrates than on the flat controls (Supplementary Fig. 2d). In addition, to further validate our findings, we imaged Paxillin dynamics in MDA-MB-231 cells shortly after plating and analysed the evolution of FA size in the initial minutes of spreading for a representative cell (Supplementary Movie 3). Initially FA sizes are small and independent of orientation, but over 30 min as the cell spreads and orients to the pattern (Supplementary Fig. 2e), the aligned FAs (≤20° deviation from direction of topographic alignment) are able to grow along the patterns to assume larger sizes, while the non-aligned ones remain confined and limited in size (Supplementary Fig. 2f). Thus, cells on aligned substrates spontaneously generate unique macromolecular arrangements of FAs that can be explained by simply assuming the spatial confinement of such complexes. As such, these findings point towards a novel and elegant mechanism of how single cells spontaneously ‘sense' and respond to ECM alignment at the molecular level.

### Constrained FA growth promotes anisotropic traction forces

**Figure 4:**
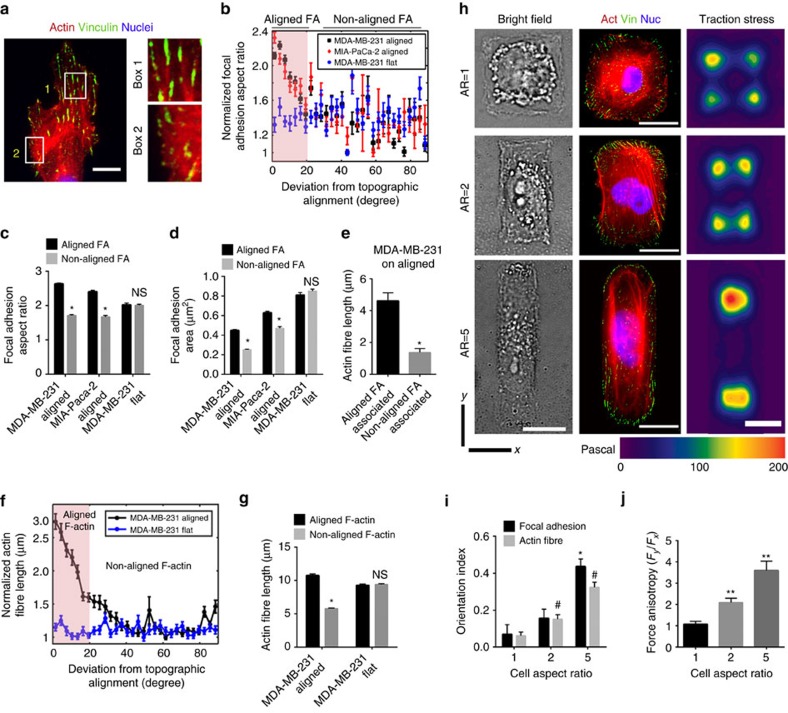
Laterally constrained adhesion growth promotes orientation and anisotropic distribution of focal adhesions and traction forces. (**a**) Fluorescence micrograph of the leading edge of a typical MDA-MB-231 cell on an aligned substrate stained for F-actin (red) vinculin (green) and nucleus (blue). Magnified Box 1 and 2 show aligned FAs connected to long stress fibre-like F-actin cables, contrary to non-aligned FAs. Scale bar, 20 μm. (**b**) Normalized FA aspect ratio of MDA-MB-231 cells (on flat and aligned substrates) and MIA-PaCa-2 cells (on aligned) as a function of the corresponding FA orientations. Data are mean ±s.e.m., aligned FA: ≤20° deviation from substrate alignment (pink region) and non-aligned FA (white region); (see Methods for additional details). (**c**,**d**) Aspect ratio (**c**) and area (**d**) of aligned and non-aligned FAs in MDA-MB-231 and MIA-PaCa-2 cells on aligned versus flat control substrates showing increased elongation and size of FAs oriented in the direction of topographic alignment (*n*>400 adhesions per cell line). (**e**) Length of F-actin fibres connected to aligned and non-aligned FAs at the cell periphery (*n*>180 fibres per group). (**f**) Normalized F-actin fibre length as a function of fibre orientations in MDA-MB-231 cells on aligned and flat substrates; data are mean ±s.e.m.; aligned F-actin: ≤20° deviation from substrate alignment (pink region) and non-aligned F-actin (white region); (see methods for additional details). (**g**) Aligned F-actin fibres are longer than non-aligned F-actin fibres in MDA-MB-231 cells on aligned but not on flat substrates (*n*>500 actin fibres per group). (**h**) Bright-field, fluorescence micrographs and traction stress maps (*n*≥7 cells per condition) of MDA-MB-231 cells patterned on ECM-coated islands in polyacrylamide gels with increasing aspect ratios (AR) of 1, 2 and 5. Scale bar, 20μm. (**i**) Focal adhesion and actin fibre orientation indices showing increasing alignment with aspect ratio (*n*>100 adhesions per condition; *P<0.0001 between AR=5 and AR=1 or AR=2 groups; and *n*>400 fibres per condition, ^#^*P*<0.0001 between AR=5 and AR=1 or AR=2 and ^#^*P*<0.01 between AR=1 and AR=2 groups by ANOVA). (**j**) Force anisotropy in long axis (*y*) versus lateral (*x*) directions with increasing aspect ratio (*n*>7 per condition; **P<0.01 for each pairwise comparison by Kruskal–Wallis test). Data in **c**–**e**,**g**,**i**,**j** are mean±s.e.m., *P<0.0001, NS: no significance (unpaired *t*-test).

With the constraint of lateral confinement, it follows that the formation and growth of FAs on aligned substrates should lead to an anisotropic size distribution of adhesions. In other words, aligned FAs would be larger and more elongated than non-aligned ones, as suggested by imaging vinculin-stained cells (Fig. 3a, Fig. 4a) and live cell imaging of paxillin dynamics in MDA-MB-231 cells on substrates (Supplementary Fig. 2e,f, Supplementary Movie 3). Indeed, global FA analysis using our semi-automated pipeline (Supplementary Fig. 2a) of typically mesenchymal-like MDA-MB-231 and MIA-PaCa-2 cells on aligned substrates revealed a sharp drop in aspect ratio of FAs with increasing deviation of their orientation from the direction of alignment (Fig. 4b). Aspect ratios of aligned FAs (≤20° deviation from direction of topographic alignment) were ∼40% greater than those of the non-aligned ones for cells on the patterned substrates (Fig. 4c). In contrast, MDA-MB-231 cells grown on flat control substrates did not show any directionally biased FA elongation (Fig. 4b,c). Likewise, on nanopatterns, aligned FA complexes were 25–50% larger in area than their non-aligned counterparts, while on flat surfaces FA size was orientation independent (Fig. 4d). Moreover, analysis of FA morphology from long-term live cell paxillin imaging of single cells on a patterned substrate indicates that aligned FAs remain consistently larger in size than non-aligned ones (Supplementary Fig. 3a).

Imaging of cells co-stained for vinculin and F-actin demonstrated that larger, more elongated aligned adhesions tend to be associated with longer stress fibre-like F-actin structures as compared to the smaller, less elongated non-aligned ones (Fig. 4a,e), consistent with current understanding of the relationship between stress fibre and FA assembly^42^,^43^. We developed a semi-automated pipeline integrating Fiji, a Curvelet transform fibre extraction algorithm (CT-FIRE) and Matlab for high-throughput global F-actin fibre morphology analysis (see Methods). These analyses revealed an F-actin size anisotropy with orientation (Fig. 4f,g), similar to the size anisotropy of FAs. Aligned actin fibres, primarily associated with aligned FAs were longer than the non-aligned fibres connected to smaller and less elongated laterally oriented FAs (Fig. 4a,e–g). Indeed examination of a typical, single MDA-MB-468 cell over time, probing its environment, demonstrates generation of lateral protrusions associated with small, non-aligned FAs (Supplementary Movie 4). However, these smaller adhesions are typically short-lived and lead to frequent retraction of the lateral protrusions. In contrast, the aligned adhesions, which are larger, more elongated and associated with longer stress fibres (Fig. 4a–e) are found to be more stable (Supplementary Fig. 3b) and hence able to sustain cell polarization along the patterned substrates. This is certainly consistent with our findings on the stability and angular constraint of lamellipodial protrusions during contact-guided migration (Fig. 2e–h, Supplementary Fig. 1b,c). Indeed, adhesion dynamics in a typical single cell demonstrate the association among the differential stability of large, aligned FAs and smaller, lateral FAs and cell elongation along the nanopatterns (Supplementary Fig. 3c). Consistent with these findings, analysis of FAs and actin fibres in MDA-MB-231 and MIA-PaCa-2 cells on aligned substrates shows that they are oriented tightly in the direction of ECM alignment in contrast to random distributions on flat substrates (Supplementary Fig. 3d), quantified by using orientation indices similar to the COI (Supplementary Fig. 3e,f). Certainly, such alignment of FA and F-actin is consistent with a number of previous reports on cell guidance by topographic alignment^23^,^25^,^44^,^45^,^46^. However, here we demonstrate how such adhesion and cytoskeletal arrangements arise spontaneously simply by geometric confinement of FAs. It is also important to recognize that cells aligned by other methods such as confinement in channels may possess aligned FA and F-actin without the orientation-dependent size distribution, since the latter is a unique feature borne out of the geometric confinement of FAs when cells encounter aligned architectures that provide contact guidance.

Focal adhesion size, maturation and orientation are known to influence, and be influenced by, traction forces that regulate cell shape, orientation and motion^7^,^47^,^48^,^49^. Based on the current knowledge in the field, we hypothesized that the biased orientation of FAs and F-actin fibres results in anisotropic traction forces that sustain cell polarization along aligned architecture. To test this hypothesis, we determined traction stress distributions from single cells with defined shape, adhesion and F-actin orientations that mimic morphologies resulting from contact guidance. Using micropatterning techniques, MDA-MB-231 cells were patterned on ECM-coated polyacrylamide gels to provide a platform facilitating alignment of FAs and stress fibres as cell aspect ratio increases from 1 to 5 (Fig. 4h,i). Consistent with our hypothesis, traction force microscopy reveals that FA alignment and accompanying stress fibre alignment results in anisotropic traction stress generation (Fig. 4h–j), with higher traction forces corresponding to larger FAs and corresponding to increasing FA alignment. Force anisotropy, defined as the ratio of forces along the direction of alignment to its orthogonal direction, increases more than threefold in cells that mimic the elongated phenotype associated with FA, actin alignment and contact guidance (Fig. 4j). Consequently, these data suggest that an increased number of bigger and longer FAs in the direction of substratum alignment produce anisotropic forces that dictate the orientation and directional persistence of single migrating cells, consistent with reports on contractility-dependent regulation of contact guidance in other cell types^30^,^50^. Thus, based on the cumulative evidence presented, our model for cellular sensing of submicrometre to micrometre-scale contact guidance cues is as follows: physical constraints of the topography limit FA growth in lateral directions leading to directional maturation and orientation of FAs along substratum guidance cues, which is associated with aligned and anisotropic F-actin organization resulting in anisotropic traction forces that bias polarization and migration of cells along aligned substrates.

### Disrupting the contact guidance response

**Figure 5:**
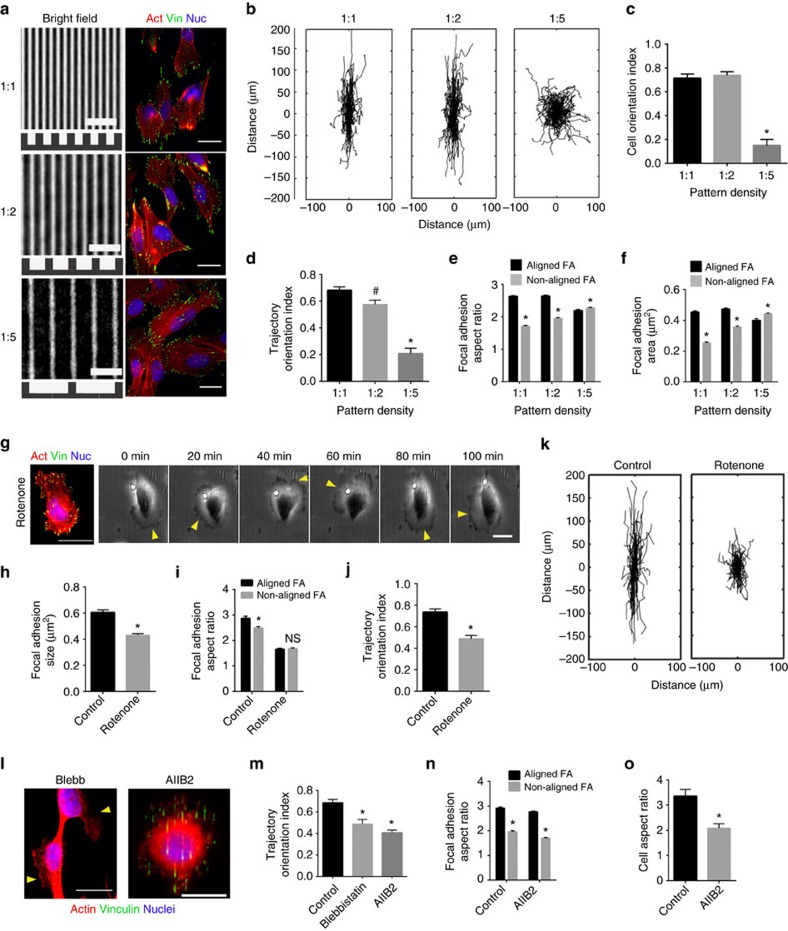
Relaxing constraints of focal adhesion growth or perturbing the focal adhesion-actin linkage diminishes contact guidance. (**a**) Nanopatterned substrates with 550 nm ridge width but varying groove widths either equal (1:1), twice (1:2) or five (1:5) times that of the ridge (scale bar, 5 μm); fluorescence micrographs showing F-actin (red), vinculin (green) and nuclei stained MDA-MB-231 cells on the corresponding patterns (scale bar, 20 μm). (**b**) Migration trajectory maps of MDA-MB-231 cells on nanopatterned substrates showing a profound decrease in directed migration with decreasing pattern density. (**c**,**d**) Cell (**c**) and trajectory (**d**) orientation indices for MDA-MB-231 cells on nanopatterned substrates with varying pattern density (**c**: *n*>100 per group; **d**: *n*>130 per group; *P<0.0001 between 1:1, 1:5 and 1:2, 1:5; ^#^*P*<0.05 between 1:1 and 1:2 by Kruskal–Wallis test). (**e**,**f**) FA size anisotropy quantified by elongation (**e**) and area (**f**) showing a reduction in the orientation-dependent FA distribution as the pattern density decreases (*n*>700 per group). (**g**) Fluorescence micrograph of a typical Rotenone-treated MDA-MB-231 cell on an aligned substrate and a timelapse montage of a similar cell showing unconstrained membrane protrusions, indicated by yellow arrowheads (scale bars, 20 μm). (**h**) FA size of 10 μM Rotenone-treated MDA-MB-231 cells is lower than the control on aligned substrates (*n*>350 per group). (**i**) FA aspect ratio showing a reversal of alignment-dependent FA elongation in Rotenone-treated MDA-MB-231 cells (*n*>350 per group). (**j**,**k**) Trajectory orientation indices (**j**) and trajectory maps (**k**) of MDA-MB-231 cells migrating on nanopatterned substrates with or without Rotenone treatment (*n*>90 per group). (**l**) Fluorescence micrographs of MDA-MB-231 cells on aligned substrates stained for F-actin (red), Vinculin (green) and nuclei (blue) showing shifts in phenotype with treatment (50 μM Blebbistatin and 10 μg ml^−1^ AIIB2) to disrupt cell traction force; yellow arrowheads indicate lateral membrane protrusions. Scale bar, 20μm. (**m**) Quantification of trajectory orientations for control, Blebbistatin and AIIB2-treated MDA-MB-231 cells (*n*>100 per group; *P<0.0001 between each group and the control by Kruskal–Wallis test). (**n**) FA aspect ratio of control and AIIB2-treated groups demonstrate similar orientation-dependent size distribution (*n*>400 per group). (**o**) Aspect ratio of control versus AIIB2-treated cells (*n*>40 per group, *P<0.0001). Bar graphs represent mean±s.e.m. *P<0.001, NS: no significance (unpaired *t*-test with Welch's correction for **e**,**f**,**h**,**i**,**n**; Mann–Whitney test for **j**,**o**).

Since data here show that contact guidance is a consequence of anisotropic FA maturation and orientation driven by spatial confinement, we hypothesized that relaxing FA confinement would disrupt the contact guidance response. This can be attained in two ways: by increasing the pattern dimensions or by reducing FA size. To achieve the first, we generated nanopatterned substrates with 550 nm ridge width but varying groove width or spacing between ridges (that is, varying the pattern density=ratio of ridge width to groove width; Fig. 5a). We utilized pattern densities of 1:1, 1:2 and 1:5, corresponding to groove widths of 550, 1,100 and 2,750 nm, respectively, where the 1:5 condition is sufficient to allow formation of large FAs (with lengths ∼2–3 μm, Supplementary Fig. 2d) with minimal orientation constraints (Fig. 5a). Consistent with our hypothesis, a marked decrease in guided cell orientations and trajectories (Fig. 5b–d) is observed as the pattern density is reduced from 1:1 to 1:5. It is striking to note that relaxing the geometric constraints on FAs to accommodate large adhesion formation without biased orientations effectively eliminates the directional response to contact guidance, in line with our model of the biophysical orchestration of this process. Further, a concomitant reversal of the anisotropic FA elongation, size and F-actin length distributions (Fig. 5e,f, Supplementary Fig. 4a) were observed with decreasing adhesion confinement along with overall decrease in the alignment of adhesions and actin fibres (Supplementary Fig. 4b,c). In addition, MIA-PaCa-2 cells also display similar reversal to almost random orientation and migration when encountered with the 1:5 pattern density aligned substrates (Supplementary Fig. 4d–g). Our data, is consistent with previous work by Kim *et al*.^23^ that shows differences in the degree of directed fibroblast migration with more sparse gradient substrates, but further explains the biophysical mechanisms underlying such observations and suggest that targeting and re-engineering the ECM in tumours to reduce the density of collagen fibre patterns may be an effective strategy to slow metastatic dissemination.

As a second approach to alleviate FA confinement independent of the substrate architecture, we globally reduced FA size in MDA-MB-231 cells using rotenone, a mitochondrial electron transport chain inhibitor that is known to reduce FA size^51^, without directly affecting the known pathways that regulate directional migration. Indeed, cells treated with rotenone had ∼30% lower FA size (Fig. 5g,h), with concomitantly reduced adhesion anisotropy (Fig. 5i). Consistent with our hypothesis, relaxing the constraints on FA growth by globally reducing FA size led to less oriented (Supplementary Fig. 4h) and less elongated cells with reduced lamellipodial constraints (Fig. 5g) and significantly lower directionality of migration (Fig. 5j,k). Combined together, these data further validate our model and suggest that the ratio of FA size and limiting pattern dimension is a critical regulator that determines contact guidance.

Our model also suggests that essential to the cellular sensing of aligned topographies is the association of confinement-driven FA anisotropy with F-actin and traction force anisotropy. We performed a series of focused experiments to disrupt this linkage and hypothesized that it would reduce cell guidance on the patterns. First, we attenuated the anisotropic traction by globally reducing force transmission to the substrate. Reducing myosin-regulated contractile force with Blebbistatin alters cell phenotype resulting in dendritic protrusions devoid of large FAs and distinct stress fibres (Fig. 5l). As a consequence, and consistent with our hypothesis, this leads to less directionally constrained membrane protrusions (Fig. 5l) and migration that is significantly less aligned with the patterns (Fig. 5m), a finding which was further validated in the MIA-PaCa-2 cell line (Supplementary Fig. 4i).

As a second approach to test our conclusions we disrupted adhesion dynamics since FAs play a vital role in force transmission between the actin cytoskeleton and the ECM. In particular we disrupted the dynamics of β1-integrin, a primary ligand for collagen and fibronectin expressed at high levels in both MDA-MB-231 and MIA-PaCa-2 cells. Treatment with β1-integrin function-blocking antibody (AIIB2) does not alter FA morphology (Fig. 5l) and therefore predictably preserves the orientation-dependent elongation of FAs (Fig. 5n). However, due to integrin function blocking, the FAs are not associated with aligned stress fibres, rather the F-actin morphology is altered to largely diffuse or cortical actin (Fig. 5l), consistent with previous work^52^. As a result, cells are much less elongated (Fig. 5o), suggesting a low traction state associated with a drastically reduced trajectory orientation (Fig. 5m). In MIA-PaCa-2 cells, the response to treatment with AIIB2 is quite similar (Supplementary Fig. 4i), suggesting that these conclusions may be valid for other mesenchymal-like cells as well. Therefore, force transduction through the actin-FA linkage is vital in translating the biased FA growth into a robust contact guidance response. In this context, since rotenone treatment reduces cell speed^51^ (Fig. 5k), presumably by affecting the intrinsic force or protrusion generation machinery, the latter could be another contributing factor in the observed reduction in contact guidance response due to rotenone treatment.

### Carcinoma cell phenotype dictates contact guidance response

**Figure 6:**
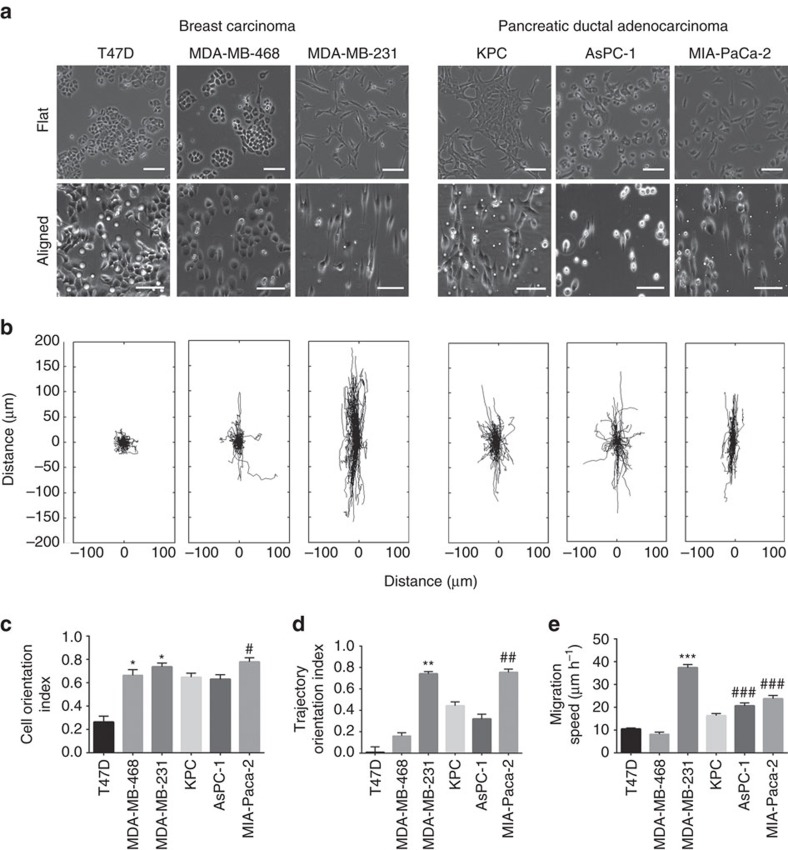
Phenotypically diverse carcinoma cells respond differently to nanoscale contact guidance cues. (**a**) Morphology of breast and pancreatic carcinoma cell lines on flat (Top) substrates showing the baseline epithelial phenotypes of T47D, MDA-MB-468, *KPC,* and AsPC-1 cells in contrast to the strong EMT phenotype of MDA-MB-231 and MIA-PaCa-2 cells. (Bottom) On aligned substrates, cells respond readily by aligning along the contact guidance cues. Scale bar, 50 μm. (**b**) Cell trajectory plots mapping migration path over time on aligned ECM demonstrate responsiveness to contact guidance and the cell line specific heterogeneity of the response. (**c**,**d**) Cell (**c**) and Trajectory (**d**) orientation indices demonstrating responsiveness to contact guidance for all cell lines and a more robust response in cells with an EMT phenotype (for **c**: *n*=70–120 cells per group; *P<0.0001 for T47D versus MDA-MB-468 and MDA-MB-231 cells; ^#^*P*<0.001 for *KPC* and AsPC-1 versus MIA-PaCa-2 cells; for **d**: *n*=90–220 cells per group; **P<0.0001 for T47D and MDA-MB-468 versus MDA-MB-231 cells; ^##^*P*<0.0001 for *KPC* versus AsPC-1 and MIA-PaCa-2 cells). (**e**) Migration speed of each cell line on aligned topography determined from the PRWM showing the highest speed in cells with an EMT phenotype (*n*=50–100 cell per group; ***P<0.0001 for T47D and MDA-MB-468 versus MDA-MB-231; ^###^*P*<0.0001 for *KPC* versus AsPC-1 and MIA-PaCa-2 cells). Data in **c**–**e** are mean±s.e.m., all comparisons by the Kruskal–Wallis test.

Our findings thus far point to the regulation of contact guided migration by anisotropic cell–substratum forces arising from geometrically confined FAs. However, our investigations have been largely focused on single mesenchymal-like, carcinoma cells that have undergone EMT, where the F-actin organization closely follows the FA distribution leading to force anisotropies. Perturbing this linkage appears to weaken the contact guidance response by altering the cytoskeletal organization, suggesting that different carcinoma cell types, lying on a spectrum between highly epithelial to mesenchymal, that possess distinct cytoskeletal organization as well as modes of migration may respond differently to the same contact guidance cues. To investigate the previously unexplored effect of multiple carcinoma cell phenotypes on contact guidance response, we quantified the behaviour of a panel of diverse primary and established cell lines from breast and pancreatic carcinomas spanning classically epithelial to very mesenchymal phenotypes (Fig. 6a, Supplementary Fig. 5a,b). These cell lines were chosen specifically to represent the heterogeneous milieu of carcinoma cells that are likely to interact with aligned collagen patterns *in vivo* (Fig. 1a,d, see Methods for detailed description of cell lines). Thus while the MDA-MB-231 and MIA-PaCa-2 are mesenchymal-like cells with loss of E-cadherin and substantial vimentin expression, T47D cells represent a differentiated epithelial phenotype with no vimentin, high E-cadherin expression and suppression of other mesenchymal markers (Supplementary Fig. 5a,b). The primary *KPC* and the AsPC-1 cell line both were vimentin positive, while also expressing E-cadherin, particularly at higher levels in the former (Supplementary Fig. 5a). Among the breast cancer lines, MDA-MB-468 represents an intermediate phenotype between the T47D and MDA-MB-231 (Supplementary Fig. 5a).

Exposing the panel of cell lines to defined ECM patterns (800 nm, 1:1) clearly reveals substantial heterogeneity in the contact guidance response (Fig. 6a,b, Supplementary Movie 5). Quantitative evaluation of cell orientation demonstrates a response for all cell lines, yet cells with an EMT phenotype respond to a significantly greater extent (Fig. 6c, Supplementary Fig. 5c). Indeed, MDA-MB-231 and MIA-PaCa-2 cells display the strongest orientation along the direction of substratum alignment (Fig. 6c). Likewise, cell trajectory orientation analysis shows enhanced directed migration along the guidance cues for the mesenchymal-like cell lines compared to their epithelial counterparts (Fig. 6d). Cells exhibiting the EMT phenotype also possess higher migration speeds (Fig. 6e) and motility coefficients (Supplementary Fig. 5d) on guiding substrates, as discerned by fitting trajectories to the PRWM^33^. Therefore, the more phenotypically epithelial cell lines, while responding to contact guidance architectures, show a remarkably lower response to this physical cue than those that are strongly of a single-cell EMT phenotype. This novel, striking finding prompted us to examine the impact of phenotypic heterogeneity within each line in greater detail.

### Cell–cell contacts compete with the cell–ECM force response

The reduced response of cells with strong cell–cell adhesion phenotype and high E-cadherin expression was particularly intriguing. E-cadherin, a known intercellular force transducer^53^,^54^, acts as the glue holding multicellular carcinoma cell clusters together. We hypothesized that cadherin-rich cell–cell interactions, which are known to induce strong, normal forces across cell–cell adhesions^55^, may counteract the anisotropic forces from cell–substratum interactions on aligned substrates.

**Figure 7:**
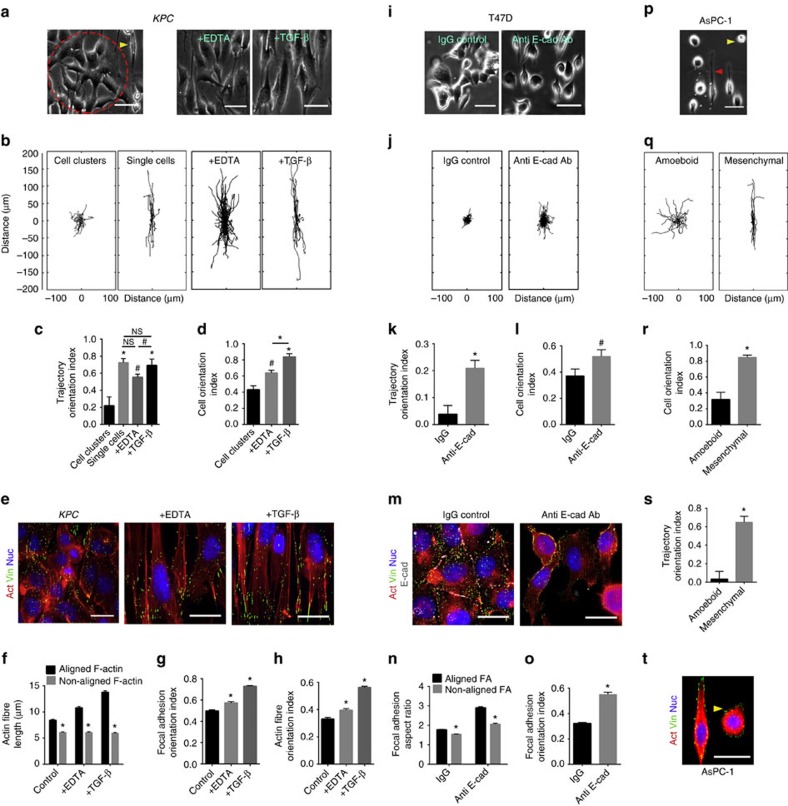
Cell–cell interactions or the amoeboid migration mode diminish directional guidance from nanopatterned cues. (**a**) Phase contrast images of *KPC* cells on patterned substrates show aligned single cells (yellow arrowhead) and randomly oriented clustered cells (red circle), while *KPC* cells treated with 2 mM EDTA or TGF-β switched to the single-cell phenotype (**b**) Trajectory maps of cells in clusters, single cells and those treated with EDTA and TGF-β. (**c**,**d**) Trajectory (**c**) and cell (**d**) orientation indices of the groups in **a**,**b**. (*n*=30–115 per group, symbols on top of columns represent comparison with the cell cluster group). (**e**) Fluorescence micrographs of *KPC* cells engaged in cell–cell clusters, EDTA and TGF-β-treated *KPC* cells on nanopatterned substrates stained for F-actin (red), Vinculin (green) and nuclei (blue) showing distinct phenotypes for single versus collective cells. (**f**) Orientation-dependent F-actin length distribution is enhanced by inducing a single-cell or EMT phenotype (*n*>1,000 per group), so are (**g**) focal adhesion (*n*>400 per group) and (**h**) actin fibre alignment (*n*>1,000 per group). (**i**) Phase contrast images of IgG control and 5 μM SHE787-treated T47D cells on aligned substrates showing clustered to single-cell phenotype shift. (**j**) Trajectory maps showing increased aligned migration in the SHE787-treated group. (**k**,**l**) Trajectory (**k**) and cell (**l**) orientation indices of the groups in **i**,**j** (*n*>70 per group). (**m**) Fluorescence micrographs of IgG control and SHE787-treated T47D cells on nanopatterned substrates stained for F-actin (red), Vinculin (green), E-cadherin (grey) and nuclei (blue). (**n**) Orientation-dependent FA elongation is enhanced by disrupting cell–cell junctions (*n*>300 per group), so is (**o**) focal adhesion alignment (*n*>700 per group). (**p**) Representative phase contrast micrograph of AsPC-1 cells on an aligned substrate showing elongated mesenchymal-like (red arrowhead) and rounded, amoeboid-like cells (yellow arrowhead). (**q**) Migration trajectory maps of the two sub-groups. (**r**,**s**) Cell (**r**) and trajectory (**s**) orientation indices of the corresponding groups in **p**,**q** (*n*>30 per group). (**t**) Fluorescence micrographs of AsPC-1 cells on nanopatterned substrates showing typical mesenchymal and amoeboid (yellow arrowhead) morphologies with staining for F-actin (red), Vinculin (green) and nuclei (blue). Scale bars, 20 μm. *P<0.001, ^#^*P*<0.05, NS: no significance (Kruskal–Wallis test: **c**,**d**; unpaired *t*-test: **f**,**l**,**n**,**o**; ANOVA: **g**,**h**; Mann–Whitney test: **k**,**r**,**s**).

To test this, we investigated the contact guidance response of the two most E-cadherin-rich cell lines viz. *KPC* and T47D. For the *KPC* line on an aligned substrate, there exists at any given time, a fraction of cells that do not form cell–cell junctions and others that are engaged in multicellular clusters (Fig. 7a). Interestingly, and consistent with our hypothesis, the single cells are highly oriented (Fig. 7a) and directionally motile (Fig. 7b,c), in contrast to the much more random behaviour of cells engaged in clusters (Fig. 7a–c). To confirm that this effect is specifically due to cell–cell adhesion, we used EDTA in growth media to deplete calcium, which is essential to the integrity of cadherin-mediated junctions^56^. Calcium chelation indeed reverts the majority of the cells into a single-cell phenotype (Fig. 7a, Supplementary Fig. 6a), with increased cell alignment (Fig. 7a,d) and enhanced directionality of migration (Fig. 7b,c) along the patterns. Alternatively, inducing a single-cell EMT phenotype via exposure to TGF-β (Supplementary Fig. 6a) results in a concomitant loss of E-cadherin expression (Supplementary Fig. 6b) and readily increases responsiveness to topographic alignment (Fig. 7a–d). In light of our previous findings, one would expect such competing cell–cell interactions to abrogate the FA and/or F-actin size anisotropy and orientation that would otherwise be observed in a single cell interacting only with the patterned substrate. In the control *KPC* group, an appreciable amount of FA anisotropy (orientation-dependent size distribution) does exist that shows a modest increase in the EDTA and TGF-β-treated groups (Fig. 7e, Supplementary Fig. 6c). However, a sharp increase in the F-actin fibre length anisotropy is observed as the cells assume a more single, mesenchymal-like phenotype (Fig. 7e,f). This is consistent with the idea that cell–cell interactions, in addition to cell–substratum interactions play an important role in organizing the actin cytoskeleton. Thus, the presence of cell–cell interactions dampens the anisotropic cell–substratum directive leading to reduced overall FA and F-actin orientations (Fig. 7g,h).

T47D cells on aligned substrates exist largely as tightly bound multicellular clusters exhibiting low motility (Fig. 6a,b), but more importantly in the context of contact guidance, low directed migration (Fig. 6d). Treatment with an E-cadherin function-blocking antibody dissociated the majority of the tightly bound clusters into single cells (Fig. 7i, Supplementary Fig. 6d). Consistent with our hypothesis and presented findings, this led to a significant increase in cell orientation (Fig. 7i,l) and directionality of migration (Fig. 7j,k). Immunofluorescence staining confirmed the presence of E-cadherin-rich cell–cell junctions in the IgG control and disruption into diffuse E-cadherin in the treatment group, sometimes accompanied by co-localization with diffuse vinculin around the cell cortex (Fig. 7m). In agreement with our model, the IgG control group exhibits a very modest FA and F-actin size anisotropy (Fig. 7n, Supplementary Fig. 6e) along with low FA and F-actin orientation indices (Fig. 7o, Supplementary Fig. 6f), which are markedly enhanced by the disruption of cell–cell interactions (Fig. 7n,o, Supplementary Fig. 6e,f). These results are in agreement with our model predictions and demonstrate further the role of cell–cell contacts in topographic sensing. Together, our present findings not only corroborate the observation by Clark and co-workers on cell–cell interaction dependent contact guidance^26^, but explain in a mechanistic framework how such variations may be orchestrated at the molecular level.

Among the three breast cancer lines, MDA-MB-468 cells represent an intermediate phenotype that form cell–cell junctions, but are not as tightly bound as the T47D cells (Fig. 6a, Supplementary Fig. 5a). Indeed, on aligned substrates, they may form cell clusters or exist as single cells (Fig. 6a) and the cell elongation in the direction of topographic alignment correlates significantly with the fraction of the cell perimeter that is free of cell–cell contacts (Supplementary Fig. 6g), further elucidating the critical role of cell–cell interactions in modulating cell guidance by topographic alignment. Closer inspection of the MDA-MB-468 cells reveal that they possess a high degree of FA size anisotropy similar to that of the MDA-MB-231 cells (Supplementary Fig. 7h,i), suggesting strong cell–ECM interactions. However, unlike typically mesenchymal-like cells, MDA-MB-468s exhibit distinct cortical actin rings that enable large, lateral stress fibre-like structures to form in association with small, laterally oriented FA complexes leading to a much-reduced F-actin size anisotropy as compared to the MDA-MB-231 group (Supplementary Fig. 7h,j). As a consequence of such cytoskeletal organization and cell–cell interactions, these cells display a much-reduced FA and F-actin orientation (Supplementary Fig. 7k,l) that ultimately contributes to their reduced responses to topographic alignment.

### Amoeboid phenotypes are less responsive to nanotopography

**Figure 8:**
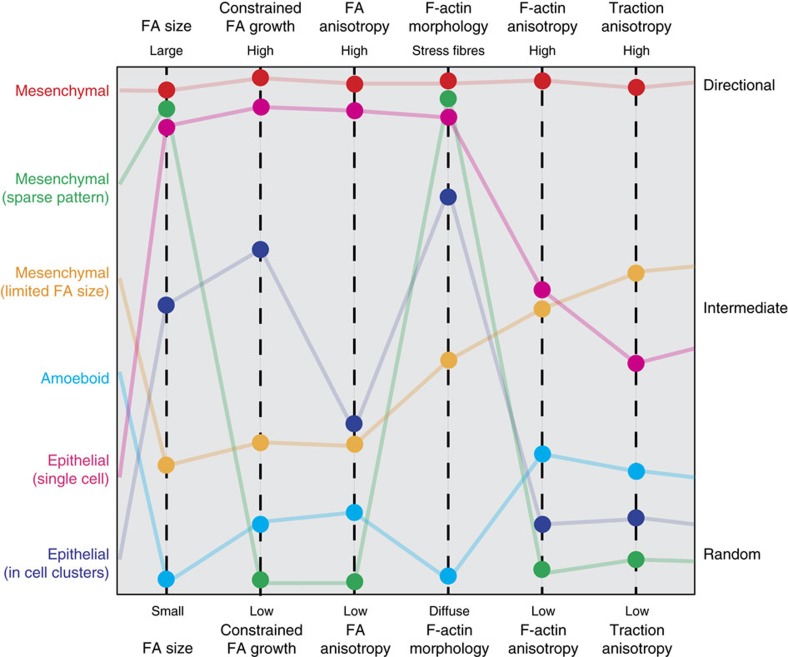
Contact guidance is driven by constrained focal adhesion growth and modulated by cell phenotype. Schematic diagram showing how disparate cell phenotypes (left most column) differ in the levels of key determinants of contact guidance response (right most column), akin to a tuning model of cell migration^58^. The relative magnitude of each parameter for a given cell type, approximated from our experimental results, is represented by color-coded dots and connected by partially blended lines as a visual aid. Each key parameter may be altered within and across different cell types (as shown in this study) by targeting intracellular and/or extracellular targets. The cumulative findings presented in this figure provide a biophysical roadmap of tunable parameters to interpret, understand and disrupt contact-guided directed migration across diverse carcinoma cell phenotypes.

In addition to epithelial and mesenchymal phenotypes, an amoeboid migration mode was observed, primarily in AsPC-1 cells, characterized by rounded motile cells with small transient protrusions (Fig. 7p, Supplementary Movies 5 and 6). A fraction of AsPC-1 cells on aligned substrates also exhibit typically mesenchymal-like phenotype (Fig. 7p). In contrast to the behaviour of phenotypically mesenchymal cells, which show robust responses similar to MDA-MB-231 and MIA-PaCa-2, directional bias of the amoeboid subpopulation is strikingly lower (Fig. 7q–s). Inspection of immunofluorescence-stained AsPC-1 cells reveal the mesenchymal-like cells predictably present with typically aligned FA and F-actin (Fig. 7t). However, the rounded, amoeboid-like cells possess a more diffuse actin cytoskeleton (Fig. 7t), similar to MDA-MB-231 cells with β-1 integrin blocking, suggesting that they represent a low traction migratory state commonly associated with amoeboid migration^57^. This finding is certainly in agreement with our model of cellular sensing of topographic patterns, which would predict that cells with smaller adhesions or low cell–ECM tractions would fail to respond robustly to the directional cues. Moreover, this data is also consistent with previously reported contact-guided amoeboid migration of *Dictyostelium*^24^. Thus, overall our findings demonstrate that contact guidance by aligned topographic cues is controlled by the balance between counteracting cell–substratum and cell–cell forces. Different carcinoma cell types with varying migration modes exist at different positions along a spectrum of relative cell–substratum and cell–cell interaction strengths, which ultimately determines the extent of their directional guidance (Fig. 8).

## Discussion

The present study represents a broad investigation spanning whole cell and molecular level interactions that mediate contact guidance of phenotypically distinct carcinoma cells, one that has been surprisingly lacking in the existing literature. We utilized nano-patterning techniques to produce controllable substrata facilitating nuanced and novel high-throughput analyses. The result of our findings is a cellular mechanism of topographic sensing that is strikingly simple and elegant, yet able to explain the diversity of responses across multiple cell phenotypes. We demonstrate that topographic patterns mimicking aligned ECM constrain FA maturation and orientation, which together with concomitant actin fibre anisotropy and orientation result in anisotropic traction forces along the direction of alignment to drive cell orientation and directional migration. While such cell–ECM forces drive single-cell migration unilaterally, cells within epithelial clusters further encounter independent competing cell–cell forces that dampen the anisotropic effect of cell–substratum interactions. Thus, the results presented here not only elucidate how cells ‘sense' ECM alignment at the molecular level, but also explain the heterogeneity of contact guidance responses from phenotypically diverse carcinoma cell populations. The latter is certainly in agreement with the notion that a particular mode of migration is ideally suited to specific microenvironmental conditions^58^.

From our conclusions on spatial constraints of adhesion growth and maturation, the ratio of adhesion size and the limiting pattern dimension emerges as a key regulator of contact guidance. Thus, for cells such as fibroblasts, which possess much larger FAs than most carcinoma cells, the range of pattern dimensions in which they respond robustly to contact guidance or the increase in pattern density required to abrogate their aligned migration are expected to scale with their FA size^23^. Our model would also predict that by reducing the depth of the patterns, one would lower the energy barrier that presumably prevents the formation of FAs spanning multiple ridges and grooves, thus making it more probable to form unconstrained FAs and consequently diminish the anisotropic response to the patterns^59^. It is also important to note that this mechanism is valid for submicrometre to micrometre-scale guidance cues such as those presented by individual collagen fibres or fibrils. However, this raises a number of interesting questions regarding the impact of simultaneous multiscale cues during guided migration. In fact, we have recently observed that while normal and transformed epithelial cells undergoing collective migration do respond to nanoscale contact guidance, consistent with observations here, cell-scale spatial confinement more strongly promotes directed collective migration^31^. Likewise, amoeboid phenotypes have been shown to respond strongly to physical confinement from channel walls or non-adhesive regions outside an ECM track or fibre^20,60^. Thus, in contradistinction to migration of cells with a mesenchymal phenotype where nanoscale contact guidance is clearly a dominant cue, directional migration of cells with active cell–cell interactions or in a low adhesion amoeboid state, while being directed by contact guidance, are likely also influenced strongly by additional multiscale spatial and chemical cues. This is consistent with the range of phenotypes and heterogeneity observed in live and archival tumour^4^,^58^ and phenotypic switching^61^ as cells navigate complex and variable 3D microenvironments in primary tumour and ectopic sites.

The finding that contact guidance is controlled by a balance of cell–ECM and cell–cell forces is an intriguing result with profound implications. For example, one can predict that reducing substrate stiffness with concomitant decrease in cell traction forces would abrogate the directional response, having a similar effect to treatment with blebbistatin or blocking integrin function. Likewise, increasing substrate stiffness may increase the directional response of clustered epithelial cells by enhancing their cell–ECM forces, although the forces across cell junctions are also likely to be altered by changing cell–substrate tractions^55^. This leads to an interesting hypothesis that prolonged exposure to stiff, aligned ECM may generate enough anisotropic traction in cells at the edge of carcinoma cell clusters to overcome their cell–cell forces and cause them to break off from a confined lesion.

In the context of disease treatment, it is always advantageous to impede the unrelenting infiltration of carcinoma cells associated with malignancy to stabilize disease while mounting a therapeutic intervention. Our findings provide important clues to identify strategies that attenuate directed migration during disease progression. Notably we find that the response to contact guidance is dominated by physical events (that is, spatial constraints on FAs and anisotropic forces). Therefore as an alternate, or perhaps complementary approach to targeting cell signalling pathways, our data suggests an opportunity to disrupt the robust directional migration programme by targeting the stroma^22^ since enhanced migration directionality and persistence on aligned ECM is abrogated most efficiently by altering the topographic pattern density. Thus, re-engineering the tumour microenvironment may be a robust strategy to slow disease and leads us to hypothesize that focused *in vivo* disruption of tumour ECM patterns and likely associated stiffness can substantially reduce the metastatic burden.

## Methods

### Cell culture

Human breast and pancreatic carcinoma cell lines (MDA-MB-231, MDA-MB-468, T47D, AsPC-1, MIA-PaCa-2) were freshly obtained from the ATCC cell bank at the start of these studies and were used within 10 passages from initial cultures, with no deviation in phenotype while remaining free of *Mycoplasma,* for all experiments. The *KPC* cell line was isolated from a pancreatic tumour in a genetically engineered *Kras*^*G12D/+*^*;Trp53*^*R172H/+*^*;Pdx1-Cre* (*KPC*) mouse (as approved by the Institutional Animal Care and Use Committee of the Univ. of Minnesota), which is a faithful mouse model for pancreas cancer^22^ and subsequently also confirmed to be free of *Mycoplasma* contamination. AsPC-1 cells were grown in RPMI 1640 media (Corning) supplemented with 10% fetal bovine serum (FBS) (Hyclone) while T47D cells were grown in RPMI 1640 supplemented with 10% FBS and 8 μg ml^−1^ insulin. All other cell lines were grown in High glucose Dulbecco Modified Eagle's Essential Medium (DMEM) (Corning) supplemented with 10% FBS.

As expected on flat substrates the breast MDA-MB-231 and pancreatic MIA-PaCa-2, both of which derive from highly invasive poorly differentiated tumours, display a strong EMT phenotype, characterized by a fibroblast-like elongated single-cell morphology and robust expression vimentin with concomitant loss of E-cadherin (Fig. 6a, Supplementary Fig. 5a). In contrast, the breast T47D and MDA-MB-468 lines present a more epithelial phenotype, grow in tightly bound clusters of cells (Fig. 6a), and are marked by the absence of vimentin while retaining E-cadherin expression (Supplementary Fig. 5a). Furthermore, T47D cells possess characteristics of well-differentiated carcinoma, form ductal structures in 3D culture, and notably lack expression of EMT markers relative to MDA-MB-231 cells (Supplementary Fig. 5a). Both the AsPC-1 cell line and a primary murine *KPC* line derive from metastatic PDA and, while expressing a number of EMT markers, including vimentin (Supplementary Fig. 5a), also form cell–cell junctions and robustly (for *KPC*) or modestly (for AsPC-1) express E-cadherin (Fig. 6a, Supplementary Fig. 5b) and thus represent a more epithelial phenotype among the PDA cells.

Stable GFP expression was induced in some of the cell lines using GFP-lentivirus particles (Gentarget) and subsequent positive selection using puromycin (Invivogen). GFP^+^ cells were amenable to high-throughput semi-automated tracking. Transient and stable expression of Paxillin-GFP was induced in MDA-MB-231 and MDA-MB-468 cells by 24–48 h incubation with Lenti-Brite lentivirus particles following the manufacturers protocol (Fisher Scientific). MDA-MB-468 cells were particularly suited for live cell imaging of FA dynamics since they possess conspicuous punctate FAs and display intermediate mesenchymal and epithelial characteristics on aligned substrates.

Cell–cell junctions in *KPC* cells were disrupted by adding 2 mM EDTA (Corning) to serum free media 4 h prior to imaging. EMT was induced by growing *KPC* cells in media supplemented with 5 ng ml^−1^ TGF-β (R&D Systems) for 4 days before plating onto substrates for experiments where the dose was maintained. E-cadherin function was inhibited in T47D cells by adding 5 μM E-cadherin function-blocking antibody (SHE78-7, Thermo Fisher) for 36–48 h before plating on substrates and subsequent imaging during which the dose was maintained. A mouse IgG antibody (SA-1-12079, Thermo Fisher) was used as the control. MDA-MB-231 cells were treated with 10 μM Rotenone (Sigma) for 4 h to reduce FA size. β1 integrin dynamics were inhibited in MDA-MB-231 cells and MIA-PaCa-2 cells by adding the function-blocking antibody AIIB2 (Development Studies Hybridoma Bank, Iowa) at concentrations of 10 μg ml^−1^ and 20 μg ml^−1^ respectively for 3 h prior to imaging. Exposure to 50 μM Blebbistatin (Sigma) for 3 h was used to inhibit myosin II in MDA-MB-231 and MIA-Paca-2 cells.

### Imaging and analysis of live tumours

Multiphoton laser-scanning microscopy images from mammary carcinomas have been previously described^16^ and are used here with permission. Pancreatic tumours from *KPC* mice that faithfully mimic human PDA with genetically engineered ZsGreen1 fluorescent protein (LSL-ZsGreen1 in the ROSA26 locus; Jackson Labs) expressed specifically in the pancreatic carcinoma cells were used for multiphoton laser-scanning microscopy imaging (as approved by the Institutional Animal Care and Use Committee of the University of Minnesota). Freshly explanted tumour sections were imaged on a custom-built multi-photon laser-scanning microscope (Prairie Technologies/Bruker) using a Mai Tai Ti:Sapphire laser (Spectra-Physics) to simultaneously generate MPE and SHG to visualize cells and collagen respectively at an excitation wavelength of 880 nm.

### Nanopatterned substrates

The nanopatterned substrates were fabricated out of Norland optical adhesives (NOAs) (Norland) which are optically clear, liquid adhesives that quickly cure when exposed to long wavelength ultraviolet light. The master designs varied according to the experimental requirements. We used NOA73 (shear modulus ∼3.67 MPa) and masters with 800 nm ridge width and spacing for most of the experiments in this study. The shear modulus was calculated from elastic moduli of the different NOAs assuming the Poisson ratio for the material to be 0.5. These conditions mimic the properties of collagen fibres under tension that are known to have moduli on the order of MPA to GPA^32^,^62^,^63^ but not the low load regions of the stress–strain behaviour that demonstrate substantially lower moduli due to the nonlinear strain-stiffening nature of collagen. To change the groove and/or ridge width of the patterns, separate silicon masters with the appropriate designs were employed.

To fabricate a sheet-type mould, the liquid was drop-dispensed onto a silicon master pattern and then a flexible, transparent polyethylene terephthalate film was brought into contact with the liquid mixture. The liquid was spread manually with a roller. Subsequently, it was exposed to ultraviolet light (*l*=200–400 nm) for 12 s. through the transparent backplane (dose=100 mJ cm). After ultraviolet curing, the mould was peeled off from the master pattern and additionally cured overnight to terminate the remaining active acrylate groups on the surface. This step creates a master that can be used to fabricate the substrate. The anisotropic substratum made of NOA 73 was fabricated on a glass coverslip using ultraviolet-assisted capillary force lithography (Fig. 1f). The glass coverslips were plasma treated for 5 min with 100 standard cubic centimeters per minute of O_2_ at 100 W and at 5 × 10^−2^ Torr to promote adhesion. They were then brushed with glass primer provided by Minuta Tech, to promote additional adhesion with a small paintbrush and then left until the liquid had dried. NOA 73 was drop-dispensed onto the glass coverslip and the master was then placed on top of the coverslip making sure that the pattern covers the glass. The drop was spread manually with a handheld roller on the polyethylene terephthalate film master side and cured for 12 s. After curing, the mould was peeled from the substratum using sharp tweezers and the completed nanopatterned substrates were cured overnight.

### Live cell imaging and quantitative analysis of cell migration

The substrates were sterilized and surface-modified using a plasma cleaner (Harrick Plasma) and then collagen-coated with 100 μg ml^−1^ rat tail collagen (BD Biosciences) in ddH_2_O for 4–6 h at 37 °C. Carcinoma cells were plated on the collagen-coated nano-patterned substrates at a density of 1.0–2.0 × 10^5^ cells per substrate and allowed to adhere and spread overnight before imaging. Single time point phase contrast images of live cells were captured using the Evos XL Core Cell Imaging System (Thermo Fisher). Cell migration data was collected by imaging at 10 min intervals over 3–4 h in an inverted Olympus IX81ZDC spinning disk confocal microscope (Olympus) under controlled temperature and pH conditions. Timelapse images were post-processed in Fiji (NIH) and cell tracking was performed using CellProfiler^64^ (for GFP^+^ cells) or using the Manual Tracking or Trackmate Plugin in Fiji. The method of overlapping intervals^65^ was used to fit the cell trajectories to the PRWM^10^ using MATLAB (Mathworks) to interface with the cell tracking output. Briefly, the mean squared displacement (MSD) for a cell for given time interval *t*_*i*_ was obtained from the average of all squared displacements *x*_*ik*_ such that


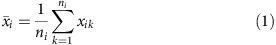


and


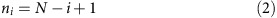


where *n*_*i*_ is the number of overlapping time intervals of duration *t*_*i*_ and *N* the total number of time intervals for the experiment. Mathematically, the PRWM can be written as


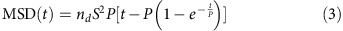


where *S* is the migration speed and *P* is the persistence time. The motility coefficient is given as


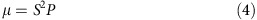


where *n*_*d*_ is the dimensionality of the random walk (in this case *n*_*d*_=2).

To quantitatively capture the biased orientation of cells, a metric termed as the COI was used. If the long axis of the ellipse that best approximated the shape of a given cell made an angle *α* (in degrees) with the axis of topographic alignment of the substrate, then the COI was defined as


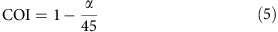


Thus, the index has an average value of 0 for a randomly oriented distribution of cells (where there is an equal probability of the deviation *α* to take any value from 0 to 90) and a maximum value of 1 for a cell which is perfectly aligned with the nanopatterns (*α*=0). Similarly to quantify how well the migration trajectories adhered to the contact guidance cues, we used the ratio of the time-averaged spread of trajectory points in the direction of alignment to that in the orthogonal direction as an estimate of the average deviation of the trajectory from the direction of alignment. First, we translated the coordinates of each trajectory point so that its position at time *t*=0 is the origin. For these transformed coordinates, we calculated the parameter


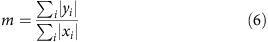


where (*y*_*i*_*, x*_*i*_) denote the coordinates of a cell in the *i*th time point along and perpendicular to the axis of alignment respectively. The metric *m* is thus a measure of the average slope of each trajectory and can be scaled similar to the COI using a simple transformation. The trajectory orientation index is thus defined as


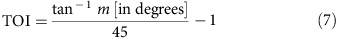


whose value is 0 for a population of cells with perfectly random trajectories and 1 for perfectly linear cell trajectories along the axis of alignment. Note that for all orientation indices calculated similarly, individual values can range from −1 to 1 and the value 0 is obtained for a perfectly random distribution only, which is seldom the case in an experimental setup. Therefore, orientation indices may assume slightly positive or negative values for such real populations of cells, especially on isotropic substrates. However, since the reference axis for the cells on flat substrates is chosen arbitrarily, for simplicity one can choose an axis that yields a positive mean value of the orientation index without loss of generality.

### Analysis of various phenotypes and cell morphology

For analysis of migration of cell clusters versus single cells, all the cells within a particular field of view that showed cell–cell connections or didn't respectively for the entire duration of the video were analysed. Data from several such fields of views across multiple experiments were pooled in the final analyses. Similar criteria were employed for analysing migration of amoeboid versus mesenchymal phenotypes. The identification of the two phenotypes was based on cell area which were vastly different (see Fig. 7t) and by the rounded morphology of the amoeboid-type cells. Analysis of cell morphology was done manually in Fiji to obtain cell area, cell aspect ratio, orientation. All cells fully within a given field of view were analysed and several such fields of view over multiple experiments were pooled in the final analyses. For the analysis in Supplementary Fig. 6g, the percentage of cell perimeter free from cell–cell adhesions was calculated for each cell and the product of its aspect ratio and the COI was used as a measure of cell elongation in the direction of topographic alignment.

### Measurement of lamellipodia dynamics

Lamellipodia dynamics were obtained from time lapse images of MDA-MB-231 and MIA-PaCa-2 cells at higher magnification, similar to a previously reported method^66^. Briefly, to capture the direction of a lamellipodial protrusion, a straight line was drawn from the approximate centre of the cell body (portion of the cell excluding the lamellipodia) to the centre of the region of the cell membrane forming the leading edge of the lamellipodia. The angle of that line to the horizontal was taken as the direction of the given protrusion. While tracking lamellipodia, only broad, flat membrane protrusions were considered. Other smaller, more irregular protrusions—such as dendritic extensions were omitted from the analysis. To evaluate lifetime, we calculate the number of time points a lamellipodia existed before being retracted (in most cases) or converted into a different type of membrane extension. For lamellipodia that existed for four or more time points, we calculated the standard deviation of the angles of the given lamellipodia during the time period of its existence as a measure of the angular spread of individual lamellipodia (Fig. 2h, Supplementary Fig. 1c). This metric serves as additional quantification of the angular distribution or range of confinement of such protrusions.

### Analysis of actin fibres and focal adhesion complexes

To analyse focal adhesion complexes, immunofluorescence images of vinculin staining were first background corrected using Fiji to enhance the punctate regions of focal adhesion clustering and eliminate diffuse background signals. CellProfiler was used to identify and extract morphometric parameters such as length, width, orientation, area and so on of such complexes from these images, before post-processing in MATLAB (Supplementary Fig. 2a). FAs from live cell imaging of GFP-Paxillin expressing cells were analysed manually using Fiji to obtain aspect ratio, orientation, area and other morphological parameters. Actin fibre analysis was performed using the Curvelet Transform Fibre Extraction (CT-FIRE: loci.wisc.edu) programme, designed to identify and analyse fibrillar structures such as collagen^67^. The focal adhesion and actin orientation indices share the same concept as the COI. Similar to COI, if *φ* is the angle made by the major axis of the ellipse that best approximates a given focal adhesion to the direction of topographic alignment, the focal adhesion orientation index (FOI) will be given by


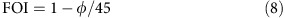


For an actin fibre, its angle of deviation from the direction of alignment is used to calculate the actin fibre orientation index in identical manner. Like the COI, these orientation indices theoretically assume values of 0 and 1 for random and perfectly aligned distributions respectively. Actin fibre lengths associated with aligned and non-aligned FAs were manually measured from phalloidin-stained micrographs using Fiji. Only distinct F-actin fibres, extending from peripheral FA complexes were used in this analysis.

When plotting focal adhesion aspect ratio (or area) with angle of deviation (Fig. 4b,c), we divided the angular spread into bins spanning 3° and plotted the mean adhesion aspect ratio (or area) centred at the midpoint of each bin along with the s.e.m. as the error bar. Actin fibre lengths as a function of angle of deviation from the direction of alignment were plotted identically (Fig. 4f). For analysis of focal adhesion size and elongation anisotropy, adhesion complexes oriented ≤20° from the direction of alignment were considered as ‘aligned FAs' and the rest ‘non-aligned FAs'. Similarly, for analysis of actin fibre length anisotropy, actin fibres oriented ≤20° from the direction of alignment were considered as ‘aligned F-actin' and the rest ‘non-aligned F-actin'.

Focal adhesion lifetimes for aligned and non-aligned FAs were calculated simply by measuring how long a particular adhesion complex, as indicated by live cell imaging of GFP-Paxillin transfected cells at 2–3 min intervals, exists before disassembling (in most cases) or sometimes in the case of non-aligned FAs, matures into an aligned FA.

### Mathematical analysis of spatial focal adhesion patterns

Projection length of a vector (of magnitude *r*) on an axis with which it makes an angle *α* is simply *r* cos *α*. Our analysis of focal adhesion complexes approximate each adhesion by an ellipse. Thus, the projection length of an adhesion on the axis along the width of the ridges and grooves is given by


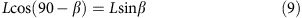


where *L* is the length of the major axis of the adhesion complex and *β* is the angle made by the major axis with the direction of substrate alignment. If the projection length of an adhesion is greater than the width of the ridge or groove in which it is situated then the adhesion is clearly not confined to that topographic unit (Fig. 3b). Thus we use the projection length to estimate the percentage of adhesions that display constrained maturation. For spacing analysis (Fig. 3e), we chose regions of parallel lines of FAs in the lamellipodia of MDA-MB-231 cells and calculated the line intensities of vinculin staining across the nanopattern arrangements using Fiji. The peak-to-peak distances calculated from these line intensity patterns using MATLAB represented the spacing between adjacent FAs. These analyses were performed on cells grown on aligned substrates with varying width of the ridges and grooves to confirm that the adhesion spacing patterns correlate directly to the length of the topographic pattern. For analysis of FA widths and lengths, large adhesions at the leading edge of a cell were analysed using Fiji with the length and width corresponding respectively to the major and minor axes of the ellipse that approximated each adhesion complex in the analysis scheme.

### Micropatterning and traction force microscopy

Micropatterned polyacrylamide gels doped with 0.2 μm fluorescent beads were prepared as previously published^68^. Briefly, polydimethylsiloxane (PDMS) stamps with 1,000 μm^2^ features and varying aspect ratios (1, 2 and 5) were fabricated using soft photolithography^69^. PDMS stamps were incubated with 100 μg ml^−1^ fibronectin (BD Biosciences) for 1 h and then dried. Stamps were placed in contact with plasma treated glass coverslips. Pre-polymer gel solution was prepared with 10/0.13/0.005% w/v acrylamide/bis-acrylamide/acrylic acid *N*-hydroxysuccinimide (Sigma) and 0.002/0.05% w/v of tetramethylethylenediamine/ammonium persulfate (Sigma). Patterned coverslips were placed onto droplets of pre-polymer solution and allowed to polymerize for 1 h at room temperature. MDA-MB-231 cells were seeded in growth media and allowed to attach for 4 h. Traction force microscopy was performed in a temperature controlled microscope chamber. A set of bright field and fluorescent images were taken prior to and after lysis of the cells with 0.5% SDS. Bead displacements were determined using an iterative particle image velocimetry algorithm implemented in Fiji^70^. Traction stresses were determined from bead displacements using an unconstrained Fourier transform traction cytometry algorithm implemented in Fiji^70^ as previously published. Traction stress vectors were discretized to 5 × 5 μm^2^ grids. Traction forces applied by the cell in the *i* axis (*x* or *y*) were calculated as


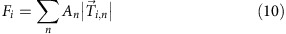


where *A* is the discretized unit surface area of the cell and 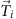 are the traction stress vectors.

### Immunofluorescence staining

For immunofluorescence staining, cells plated on flat or aligned substrates were fixed in 4% paraformaldehyde (Sigma) for 20 min, permeabilized with 0.1% Trition-X-100 (Roche) and blocked with 2% Fatty acid free-BSA (Fisher Scientific)+2.5% goat serum (Sigma), followed by incubation with mouse anti-vinculin (1:400) primary antibody (Sigma, V9131) for 40 min at room temperature, followed washing. Alexa-fluor anti-mouse secondary antibody (1:200; Life Technologies, A31571) along with 1:1,000 rhodamine phalloidin (Life Technologies, R415) and 1:10,000 bisbenzamide/Hoechst 33342 (ThermoFisher, H3570) were used as secondary agents followed by wash steps and mounting with Prolong Gold (Life Technologies). For co-staining with E-cadherin, the anti-E-cadherin antibody DECMA-1 (Millipore) was used at 1:100 concentration with an Alexa-fluor anti-rat secondary antibody (1:200, Life Technologies, A21247).

### Western blotting

Cell lysates were prepared in 1 × RIPA Lysis buffer (Millipore) supplemented with 1:100 Protease Inhibitor Cocktail (Sigma), 1:100 Phosphatase Inhibitor Cocktail 2(Sigma) and 1:100 Phosphatase Inhibitor Cocktail 3 (EMD Chemical/Calbiochem). In total, 10 μg of protein from each sample was separated using SDS–polyacrylamide gel electrophoresis, transferred to a PVDF membrane and blocked with 5% milk (Biorad). Primary antibodies used were 1:1,000 Mouse anti-E-cadherin (BD Biosciences, BDB610181), 1:500 mouse anti-Vimentin (Sigma, V5255) and 1:1,000 rabbit anti-Tubulin (Cell Signaling, 21485). HRP-conjugated anti-mouse (1:10,000) and anti-rabbit (1:10,000) secondary antibodies (Biorad) were allowed to bind before washing and probing the bands using Luminata Classico Western HRP substrate (Millipore). Unedited scans of the original blots are presented in Supplementary Fig. 7.

### Electron microscopy

The nanopatterned substrates were sputter coated with 20 nm of Gold Palladium with a Denton Vacuum Desk 3 sputter coating unit and imaged with an FEI Sirion Scanning Electron Microscope courtesy of the Molecular Analysis Facility at University of Washington, Seattle. Data for SEM of collagen fibres was previously reported^16^ and used here with permission. Analysis here was performed in the dataset to quantify the spacing between collagen fibril bundles within collagen fibres.

### Statistical analysis

Student's *t*-test was used to compare null hypotheses between two groups for normally distributed data and for large datasets (for example, FA and F-actin analyses with *n*∼500). In some cases, where clear differences in variability of the two groups exist (for example, for flat versus aligned comparisons), Welch's correction for unequal variance was used. The non-parametric Mann Whitney test was employed for non-normal or small datasets. Multiple groups were compared by ANOVA, followed by the Tukey posthoc analysis, or the non-parametric Kruskal–Wallis test with Dunn's *post hoc* testing, as dictated by the size and distribution of the data. Unless otherwise mentioned, the data analysed were pooled from many cells/field of view over several fields of view across *n*≥2 experiments. Number of data points for each experiment, the specific statistical tests and significance levels are noted in the figure text.

### Data availability

The authors declare that all data supporting the findings of this study are available within the paper and its Supplementary Information Files or from the authors upon reasonable requests.

## Additional information

**How to cite this article:** Ray, A. *et al*. Anisotropic forces from spatially constrained focal adhesions mediate contact guidance directed cell migration. *Nat. Commun.* **8,** 14923 doi: 10.1038/ncomms14923 (2017).

**Publisher's note:** Springer Nature remains neutral with regard to jurisdictional claims in published maps and institutional affiliations.

## Supplementary Material

Supplementary Information Supplementary Figures

Supplementary Movie 1 Contrasting migratory behavior of MDA-MB-231 cells on adjacent flat and aligned topographies. Scale bar=20μm.

Supplementary Movie 2 Focal adhesion dynamics of a representative MDA-MB-468 cell expressing GFP-Paxillin at the interface of flat and aligned topographies showing spontaneous confinement and alignment of adhesions in the aligned region. Scale bar=20μm.

Supplementary Movie 3 Focal adhesion dynamics of a representative MDA-MB-231 cell expressing GFP-Paxillin on an aligned substrate in the early stages of cell spreading and elongation. Scale bar=20μm.

Supplementary Movie 4 Focal adhesion dynamics of a representative MDA-MB-468 cell expressing GFP-Paxillin on an aligned substrate showing intermittent lateral probing and contrasting stability of aligned versus non-aligned adhesions. Scale bar=20μm.

Supplementary Movie 5 Cell migration of a panel of six breast and pancreatic carcinoma lines in response to aligned topographies showing the diversity in guidance responses with cell phenotype. Scale bar=50μm.

Supplementary Movie 6 Migratory behavior of a rounded, amoeboid-like AsPC-1 cell on aligned topography showing small, transient protrusions and movement orthogonal to the pattern alignment. Scale bar=20μm.
