# Supplementary material for: Anisotropic forces from spatially constrained focal adhesions mediate contact guidance directed cell migration
Source: Nat Commun. 2017 Apr 12;8:14923. doi: 10.1038/ncomms14923 (PMC5394287; doi:10.1038/ncomms14923)
Supplement: Supplementary Information — Supplementary Figures [file ncomms14923-s1.pdf]

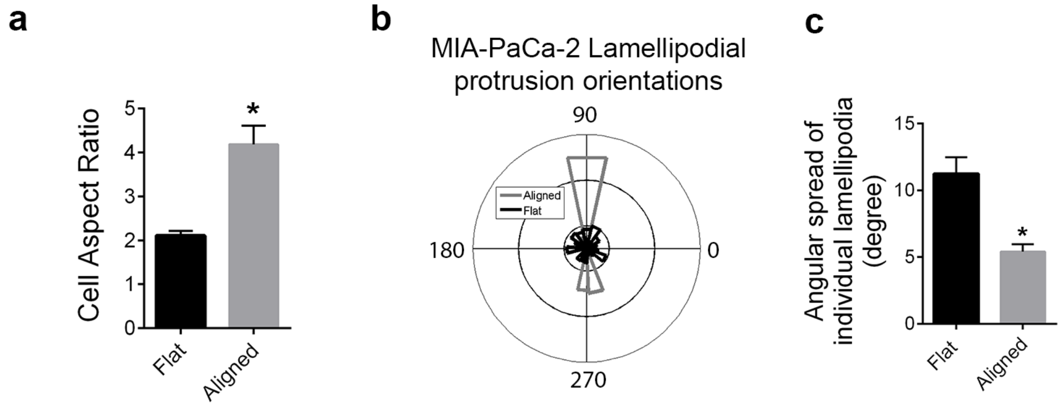

Supplementary Figure 1: Cell elongation and protrusion dynamics in response to contact guidance: (**a**) Aspect ratio of MDA-MB-231 cells on flat and aligned substrates (n>50/group). (**b**) Wind-rose plot of MIA-Paca-2 lamellipodia protrusion orientations showing highly directed protrusion dynamics on aligned ECM (n>400 lamellipodia/group). (**c**) Angular spread of MIA-PaCa-2 lamellipodia on control and nanopatterned substrates (n>40/group). Data are mean ± SEM in a and c; *p<0.001 (Mann-Whitney test).

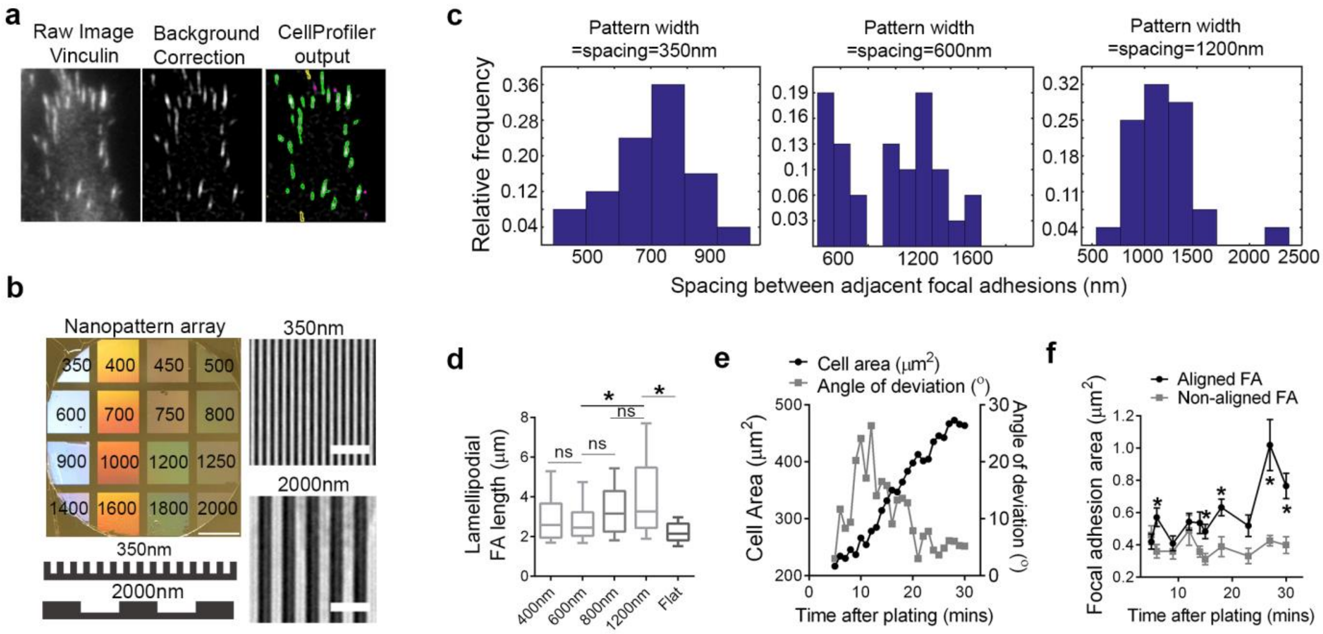

Supplementary Figure 2: Spatial confinement-driven adhesion growth and distribution on patterned substrates: (**a**) Representative images showing the platform for high throughput semi-automated analysis of FAs involving background correction of raw vinculin-stained micrographs in Fiji and subsequent identification and morphometric analysis of individual FAs (identified adhesions are outlined in green, pink and yellow outlines denote those objects discarded by size thresholding and due to touching the border of the image respectively). (**b**) Nano-patterned substrate design for multiplexed analysis of cell behavior on 16 different 1:1 ridge and groove width dimensions (numbers on the nanopattern array indicate equal ridge and groove dimensions in nm for each island). Schematic side views and bright-field top views for 350 and 2000 nm 1:1 substrates provide examples of the pattern dimensions and spacings employed here. (Scale bars=3µm and 6µm for 350nm and 2000nm bright-field images respectively). (**c**) Histogram of peak-to-peak distances between adjacent focal adhesions on a 350nm pattern spacing (*left*) displaying a unimodal distribution with median around 700nm, a 600nm pattern spacing (*middle*) with a bimodal distribution with medians around 600nm and 1200nm, and a 1200nm spacing pattern (*right*) displaying a unimodal distribution with median around 1200nm. (**d**) Length of large, aligned FAs at the leading edge of cells show a modest increase on aligned substrates with increasing pattern widths and a significant decrease on flat control substrates with medians ranging between 2-3µm across the groups (n>65/group, *p<0.001, ns=no significance by Kruskal-Wallis test; data are median with 10-90th percentile range). (**e**) Evolution of cell spread area and alignment of a typical MDA-MB-231 cell on an aligned substrate (shown in Supplementary Movie 3) minutes after plating and (**f**) area of aligned (<=20° from topographic alignment) and non-aligned FAs over time during the same period showing increasing size of the former while the latter remains constant (n=5-17 FAs/group, data are mean ±SEM; *p<0.05 by Mann-Whitney test).

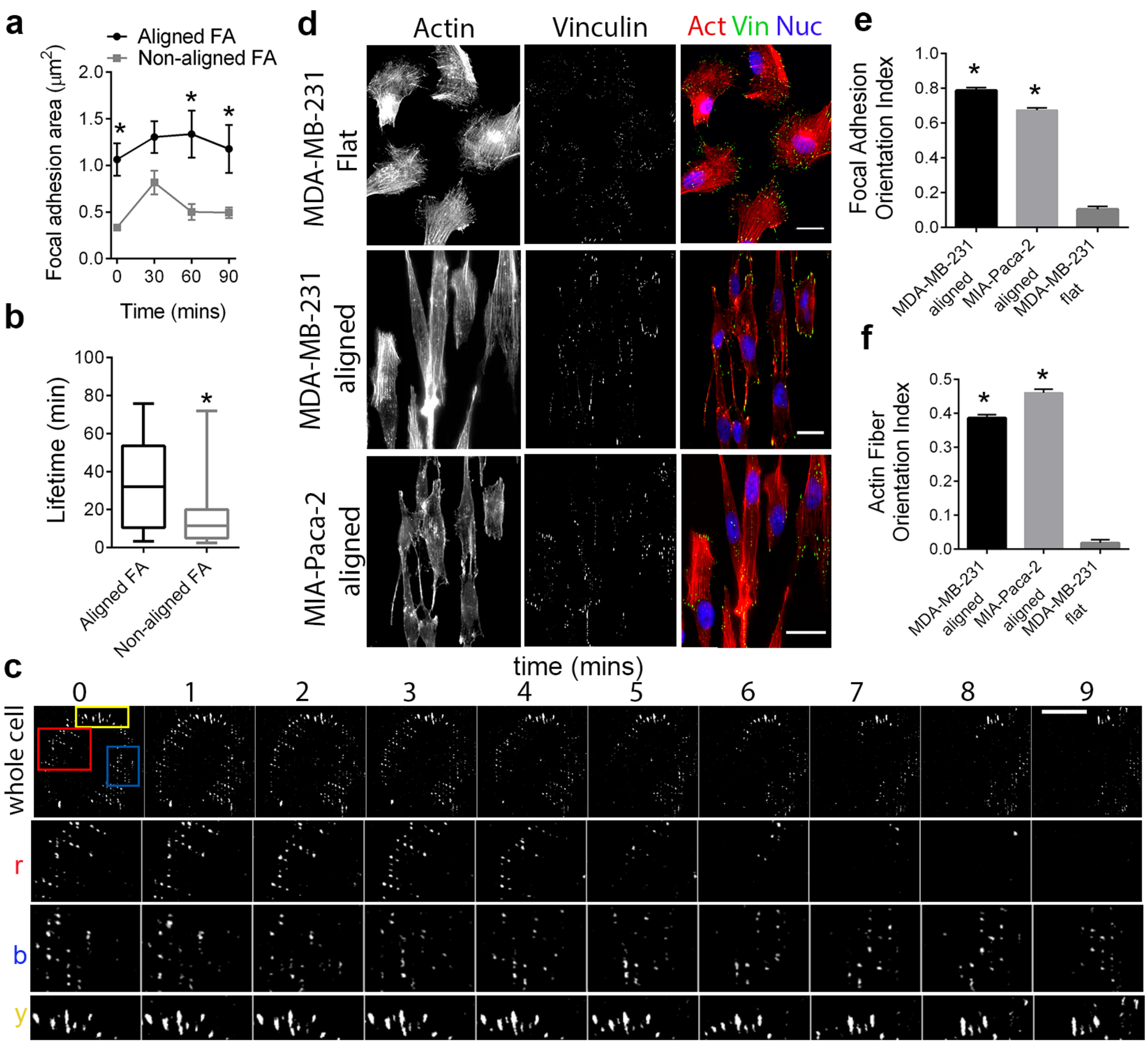

Supplementary Figure 3: Anisotropic stability and size distribution of focal adhesions and associated adhesion and F-actin alignment: (**a**) Area of aligned and non-aligned FAs over time in a typical MDA-MB-468 cell expressing GFP-Paxillin on aligned topography demonstrating that aligned FAs maintain larger area over time (n>20 adhesions/time point, *p<0.05 by Mann-Whitney test). (**b**) Lifetime of aligned and non-aligned FAs analyzed from live imaging of single MDA-MB-468 cells expressing GFP-Paxillin on aligned substrates (n=25/group, *p<0.05 by Mann-Whitney test). (**c**) Timelapse montage (background corrected) of live imaging of a representative single MDA-MB-468 cell expressing GFP-Paxillin showing a shift from rounded to elongated cell morphology accompanied by disassembly of small, non-aligned adhesions on one side (red box), maintenance of small, non-aligned adhesions on the other side (blue box), while larger aligned adhesions remain stable (yellow box). (**d**) Fluorescence micrographs of F-actin, Vinculin and nuclei-stained MDA-MB-231 and MIA-PaCa-2 cells on aligned substrates. (**e and f**) Quantification of FA orientation (e) and actin fiber orientation using Curvelet Transform analysis (f) showing alignment of FAs and actin filaments in the direction of topographic alignment (e: n>400 adhesions/group; f: >3000 fibers/group; *p<0.0001 between aligned and flat conditions by unpaired t-test with Welch's correction). Data in a, e and f are mean ± SEM, scale bars=20μm.

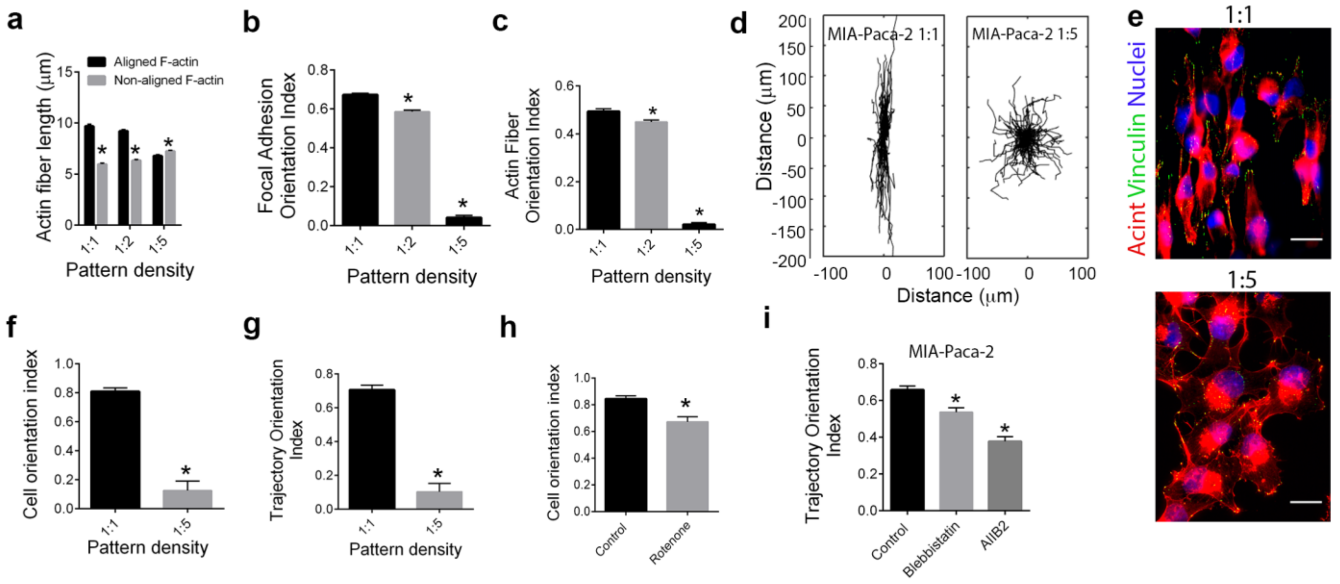

Supplementary Fig. 4: Relaxing geometric constraints to FA growth and disrupting cell-substratum force transduction diminishes contact guidance response: (**a**) Orientation-dependent F-actin fiber length in MDA-MB-231 cells is reduced as the pattern density decreases from 1:1 to 1:5 (n>1000 fibers/group, *p<0.0001). (**b and c**) Focal adhesion orientation (b) and actin fiber orientation (c) demonstrating a significant decrease in alignment in the direction of ECM as the pattern densities change from 1:1 to 1:5 (g: n>2700 adhesion/group; h: n>2500 fibers/group; g and h: *p<0.0001 between 1:1 vs. 1:2 and 1:1 and 1:2 vs. 1:5 by ANOVA). (**d**) Migration trajectory maps of MIA-PaCa-2 cells on nanopatterned substrates with differing pattern densities of 1:1 and 1:5 showing a profound decrease in directed migration as the geometric constraints are relaxed in the 1:5 case. (**e**) Fluorescence micrographs of MIA-PaCa-2 cells on 1:1 and 1:5 substrates, stained for F-actin (*red*), Vinculin (*green*) and nuclei (*blue*) showing the decrease in cell and associated actin and adhesion orientation as the groove dimension increases; Scale bar = 20µm. (**f and g**) Cell (f) and trajectory (g) orientation indices for MIA-PaCa-2 cells on 1:1 and 1:5 pattern density substrates (f: n>45 cells/group; g: n>100 cells/group; *p<0.0001). (**h**) Cell orientation index of control and rotenone treated MDA-MB-231 cells on aligned substrates (n>70/group, *p<0.0001). (**i**) Trajectory orientation indices for MIA-PaCa-2 cells treated with 50µM Blebbistatin or 10µg/ml AIIB2 on nanopatterned substrates (c: n>240 cells/group; *p<0.0001 by ANOVA). Bar graphs represent mean ± SEM, comparisons in a, f-h by unpaired t-test with Welch’s correction.

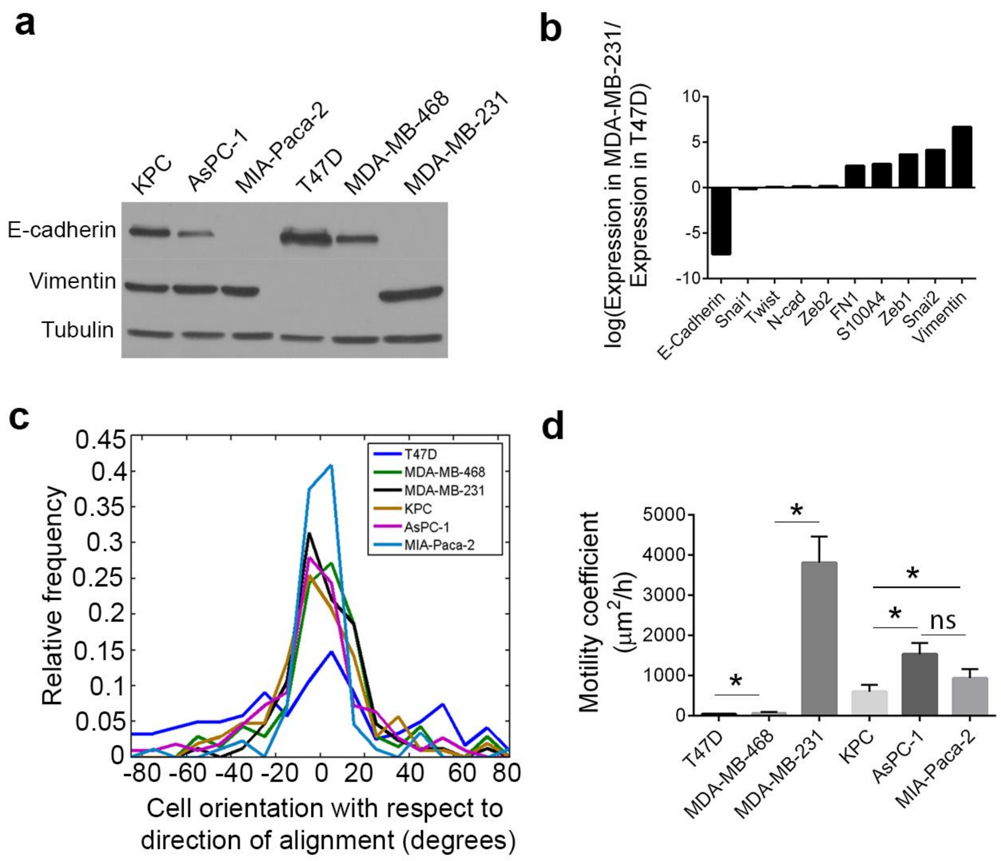

Supplementary Figure 5: Phenotypic diversity, difference in cell orientation and motility of the breast and pancreas carcinoma cell panel: (**a**) Western blot showing loss of E-cadherin expression in cells with an EMT phenotype (MDA-MB-231 and MIA-PaCa-2) along with vimentin expression in all pancreatic cancer lines and only in MDA-MB-231 cells among the breast cancer lines (See Supplementary Fig. 7 for unedited blots). (**b**) Comparison of EMT marker mRNA expression between MDA-MB-231 and T47D cells quantified from publically available gene microarray data (Accession: GSE5846). (**c**) Histograms for cell orientation for each cell line with respect to direction of substratum alignment (i.e. 0°). (**d**) Motility coefficient determined from the persistent random walk model (n=50-110 cells/group; *p<0.01 for all comparisons except AsPC-1 vs. MIA-PaCa-2 which are not significantly different by Kruskal-Wallis test), data are mean ± SEM.

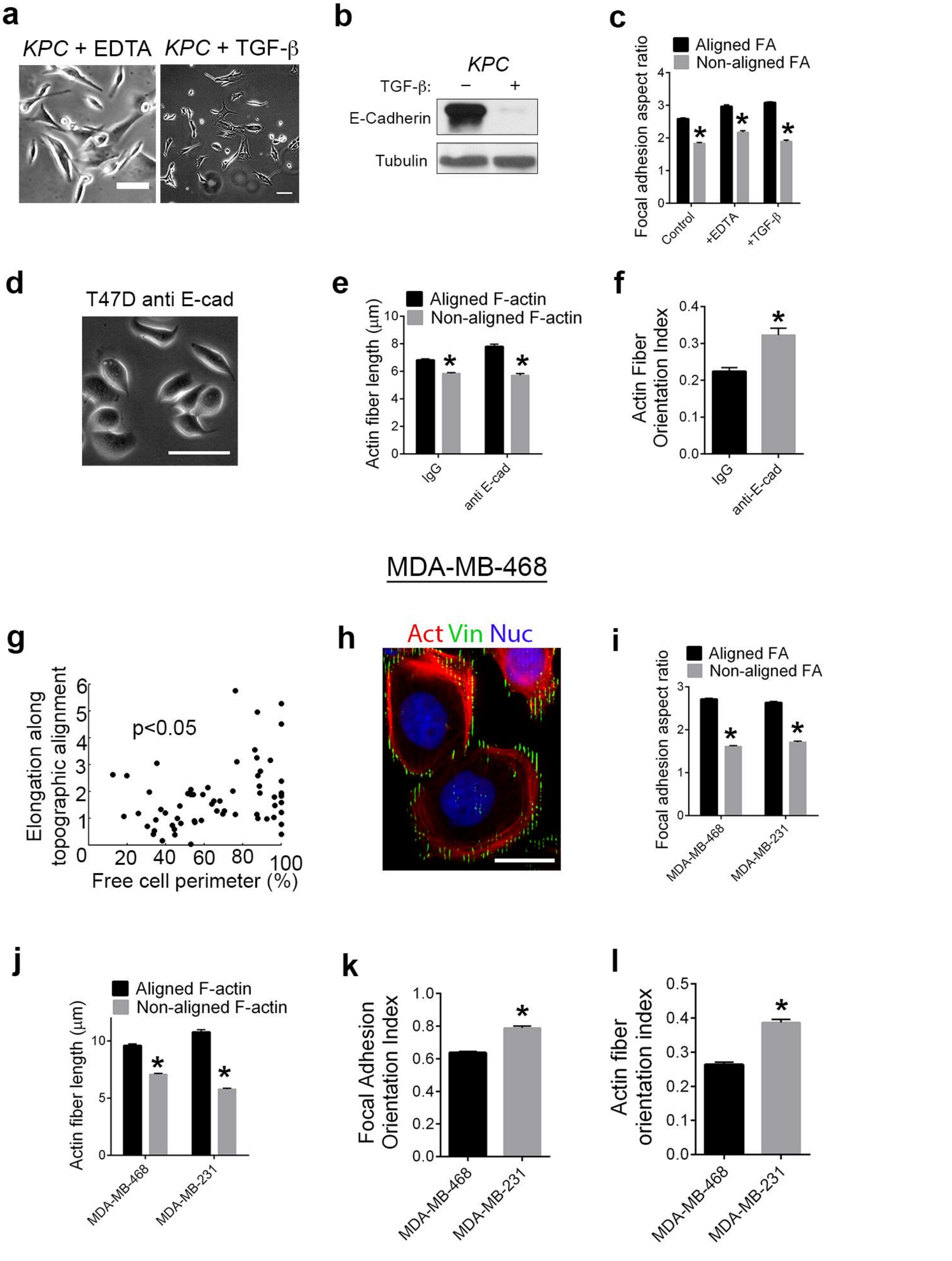

Supplementary Figure 6: Presence of cell-cell interactions or cortical actin diminish contact guidance response to nanopatterned substrates: (**a**) Phase contrast micrographs of *KPC* cells treated with EDTA and TGF-β respectively showing their predominantly single cell phenotype. Scale bars=50µm. (**b**) E-Cadherin western blot indicating the induction of the EMT phenotype in *KPC* cells treated with TGF-β on flat and aligned substrates (See Supplementary Fig. 7 for unedited blots). (**c**) Orientation-dependent FA elongation of EDTA or TGF-β-treated *KPC* cells as compared to the control (n>300/group). (**d**) Phase contrast micrograph of SHE787-treated T47D cells showing dissociation of

tight cell-cell clusters, Scale bar=50µm. (**e**) Anisotropic size distribution of F-actin fibers and (**f**) overall biased orientation on aligned substrates is enhanced in the function blocking antibody-treated group (e: n>300/group and f: n>1000). (**g**) Scatter plot showing elongation of cells in the direction of alignment (aspect ratio x cell orientation index) as a function of percentage of the cell perimeter free of cell-cell connections for MDA-MB-468 cells on aligned substrates, p-value for the null hypothesis that no correlation exists is noted. (**h**) Fluorescence micrographs of representative MDA-MB-468 cells on nanopatterned substrates stained for F-actin (*red*), Vinculin (*green*), E-cadherin (*gray*) and nuclei (*blue*) showing anisotropic FA size and cortical actin distributions. Scale bar = 20µm. (**i**) Comparison of anisotropic FA elongation in MDA-MB-468 cells with that of MDA-MB-231 cells showing similar behavior (n>400/group). (**j**) Anisotropic F-actin length distribution in MDA-MB-468 cells showing reduced anisotropy as compared with that of MDA-MB-231 cells on aligned substrates (n>700/group). (**k and l**) FA (k) and F-actin (l) are much less oriented in MDA-MB-468 cells than in MDA-MB-231 cells (n>1000/group). All bar graphs represent mean ± SEM; *p<0.0001 (unpaired t-test).

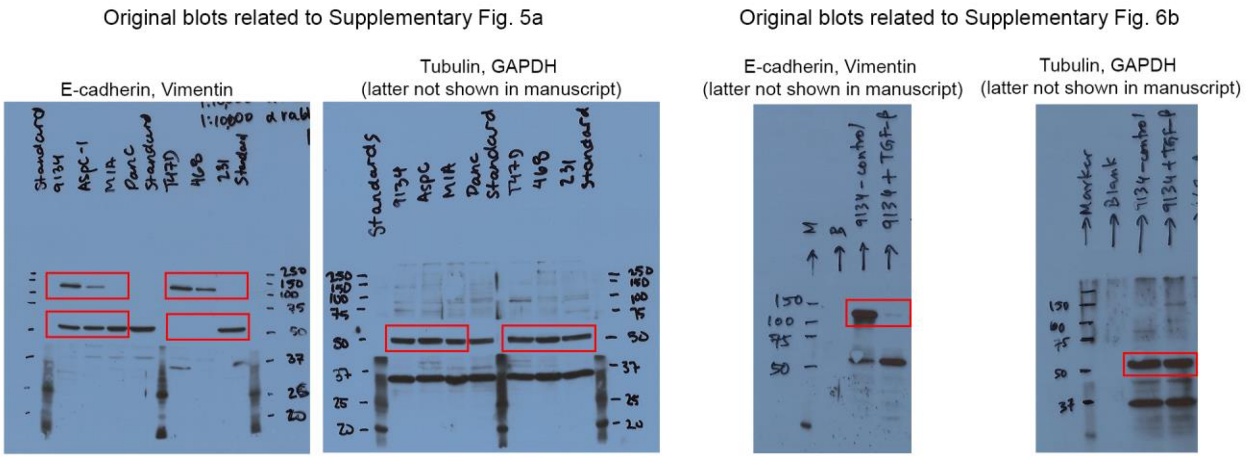

Supplementary Figure 7: Unedited original western blotting films
